# Elasticity‐Driven Nanomechanical Interaction to Improve the Targeting Ability of Lipid Nanoparticles in the Malignant Tumor Microenvironment

**DOI:** 10.1002/advs.202502073

**Published:** 2025-03-27

**Authors:** Eunhee Lee, Loi Nguyen Dang, Jinsol Choi, Haesoo Kim, Lyndon Bastatas, Soyeun Park

**Affiliations:** ^1^ Department of Pharmacy Keimyung University 1095 Dalgubeoldaero Dalseo‐gu Daegu 42601 Republic of Korea; ^2^ Premier Research Institute of Science and Mathematics, Department of Physics Mindanao State University Iligan Institute of Technology Andres Bonifacio Ave Tibanga, Iligan City, Lanao del Norte 9200 Philippines

**Keywords:** atomic force microscopy, cellular uptake, lipid nanoparticles, mechanical property, permeation

## Abstract

The mechanical elasticity of lipid nanoparticles (LNPs) is crucial to their pharmaceutical performance. This study investigates how the mechanical interactions between LNPs, target cells, and macrophages affect the internalization of LNPs into target cells at tumor sites. According to our bio‐mechanical study, drug‐resistant breast cancer cells are stiffer than sensitive ones, while invasive cells are softer; similarly, protumoral M2 macrophages are softer than M1 macrophages. Softer LNPs show increased cellular uptake in breast cancer cells and macrophages, with enhanced engulfment in invasive cells and M2 macrophages. Additionally, the presence of M2 macrophages promotes greater LNP internalization by cancer cells, facilitating the malignant and invasive nature of cancer cells. In addition, because breast cancer cells engulf LNPs via an energy‐efficient fusion pathway but LNPs in macrophages undergo clathrin‐mediated endocytosis, LNPs are internalized more into cancer cells but not into M2. In orthotopic tumor models, softer LNPs penetrate tumors quickly, enhancing suppression, whereas stiffer LNPs permeate slowly but show prolonged retention in stiffer tumors, supporting antitumor efficacy with repeated dosing. These findings underscore the importance of mechanical interactions between LNPs, target cells, and macrophages in optimizing LNP delivery systems, offering insights for more effective designs.

## Graphical abstract

The elasticity of lipid nanoparticles (LNPs) is essential to their tumor‐targeting efficacy. Softer LNPs penetrate tumors faster and more easily enter into softer cancer cells, while stiffer LNPs show prolonged retention in stiffer tumors. The presence of protumoral M2 macrophages enhances the LNPs uptake into stiffer cancer cells, facilitating malignant environments. These biomechanical interactions provide valuable insights for designing LNP‐based chemotherapeutics.

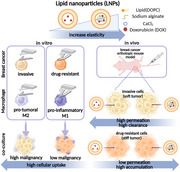

## 1 Introduction

Lipid nanoparticles (LNPs) have emerged as promising carriers of small molecules and biomacromolecules. The success of LNPs for drug delivery is intricately linked to their unique physicochemical properties. Specifically, the size and surface charge of LNPs control their pharmacokinetic behavior.^[^ ^1^ ^]^ The elastic moduli of LNPs have recently emerged as pivotal determinants of their interactions with the target cells and tissues.^[^ ^2^ ^]^ For example, a study reported that soft silica nanoparticles (NPs) were better absorbed in cancer cells but not in macrophages.^[^ ^3^ ^]^ Conversely, another study reported that soft silica NPs showed reduced cellular binding and endocytosis rates.^[^ ^4^ ^]^ Fine‐tuning the elasticity of LNPs has been shown to reduce the off‐target effect and enhance anticancer efficacy.^[^ ^5^ ^]^ Additionally, adjusting the elasticity of both lipid and polymer‐based NPs improved the half‐life, accumulation in tumor sites, and systemic circulation, ultimately affecting their antitumor efficacy.^[^ ^6^ ^]^ However, it remains unclear how the elasticity of LNPs affects their pharmacokinetic behavior and thus therapeutic applications. Therefore, more systematic investigations are required to fully understand these phenomena.

The cellular uptake of LNPs is affected by the mechanical properties of both the LNPs and cancer cells. Over the recent decades, one of the most significant findings in biomechanical research has been that cancer cells are mechanically more compliant than normal cells.^[^ ^7^ ^]^ Additionally, cancer cells dynamically adjust their elasticity in response to microenvironmental cues and chemotherapeutic treatments.^[^ ^8^ ^]^ For example, drug‐resistant ovarian cancer cells are stiffer than sensitive ones.^[^ ^9^ ^]^ Such mechanical properties of cancer cells may significantly affect the permeation of LNPs into target cells. Therefore, understanding the relationship between the cellular uptake of LNPs and cell elasticity is essential to gain deeper insights into these effects.

Furthermore, mechanical stimuli from the intricate biological milieu that LNPs encounter on their transit to target sites ultimately influence their fate. In systemic circulation and at tumor sites, LNPs interact mechanically with stromal cells and the extracellular matrix (ECM).^[^ ^10^ ^]^ Previous studies have shown that stiff substrates significantly enhance the cellular uptake of polymeric micelles in cancer cells via receptor‐mediated endocytosis, though this effect is not observed in epithelial cells or macrophages for silica NPs.^[^ ^11^ ^]^ However, polystyrene NPs showed up to 15 times higher diffusion efficiency in an aligned network of ECM with low density and stiffness.^[^ ^12^ ^]^ Furthermore, the interaction of various kinds of NPs with macrophages highly affects their delivery to target sites including the liver.^[^ ^13^ ^]^ The mechanical properties of hydrogel and gelatin NPs also affect their internalization into macrophages, similar to their effects on cancer cells.^[^ ^14^ ^]^

Macrophages are polarized into M1 macrophages by proinflammatory signals, such as interferon‐gamma (IFN‐γ) and microbial products such as lipopolysaccharide (LPS) while the M2 phenotype is activated by cytokines such as interleukin‐4 (IL‐4) and interleukin‐13 (IL‐13), exhibiting anti‐inflammatory behaviors.^[^ ^15^ ^]^ In addition to functional differences, these polarized macrophages may also differ in their mechanical properties, potentially affecting nanoparticle (NP) uptake.^[^ ^16^ ^]^ For instance, rapid internalization of stiff gelatin NPs by RAW264.7 macrophages has been reported.^[^ ^14b^ ^]^ Similarly, stiff hydrogel NPs were more efficiently engulfed by J774 macrophages.^[^ ^17^ ^]^ Conversely, Sun et al. reported a decrease in the capture of soft LNPs by RAW264.7 cells.^[^ ^18^ ^]^ These studies indicate that the triple mechanical interplay between LNPs, cancer cells, and their surrounding microenvironment is a key factor affecting the entry of LNPs into tumors. Further studies are required to confirm the role of the elasticity of NPs in their uptake by macrophages with different polarization states.

Atomic force microscopy (AFM) is widely used to study the mechanical properties of NPs and cells.^[^ ^9^, ^19^ ^]^ A simple round‐trip motion of an AFM probe over samples yields a force–distance (*f*–*d*) curve, from which the elasticity is calculated using mathematical models.^[^ ^20^ ^]^ However, the small size, heterogeneity, and extreme deformability of NPs and cancer cells pose challenges. For cells, selecting an appropriate probe and force regime is essential to avoid irreversible damage and deformation, ensuring reliable elasticity measurements.^[^ ^21^ ^]^ For NPs, the influence of hard substrates, such as mica, on which the NPs are placed for measurements, introduces additional difficulties in accurately measuring elasticity.^[^ ^22^ ^]^ To resolve the intricate features captured in *f*–*d* curves, we adopted the Capella model in our previous study,^[^ ^23^ ^]^ which accounts for the asymptotic behavior reflected in *f*–*d* curves in multilayered samples. In the study, we obtained *f*–*d* curves in the two‐dimensional array over the samples and analyzed them using the Capella model to accurately determine the elastic moduli of the inner core and outer shells of the LNPs and cells.^[^ ^19^, ^23^ ^]^

From a pharmaceutical perspective, it is crucial to understand the detailed mechanisms by which LNPs enter cells, particularly by elucidating the role of the mechanical properties of LNPs and cells in their cellular uptake. The cellular uptake of LNPs is primarily facilitated by endocytosis, a process in which cells engulf LNPs within the plasma membrane. Endocytosis pathways are classified based on energy consumption and the type of associated receptor.^[^ ^24^ ^]^ Increasing evidence suggests that the elastic properties of LNPs influence their internalization into cells.^[^ ^25^ ^]^ However, the exact correlation between elasticity and cellular uptake pathways remains inconsistent. For example, Liu et al. showed that stiffer hydrogel particles predominantly enter cells via both caveolae‐ and clathrin‐mediated endocytosis as well as macropinocytosis, whereas softer hydrogel NPs primarily utilize macropinocytosis.^[^ ^26^ ^]^ Conversely, Guo et al. reported that stiff LNPs with an alginate core are internalized via clathrin‐mediated endocytosis, while soft LNPs enter cells through both fusion and endocytosis.^[^ ^27^ ^]^ Several research groups have further investigated the different pathways experienced by stiff and soft LNPs with polymeric cores during internalization.^[^ ^27^, ^28^ ^]^ Despite these efforts, drawing a definitive conclusion on how NP stiffness controls the internalization pathways involved in cellular uptake remains premature.

In this study, we evaluated the mutual mechanical effects of LNPs, cancer cells, and macrophages on cellular uptake, internalization pathways, tumor penetration, accumulation, and tumor suppression, using both in vitro and in vivo breast cancer models. To achieve this, we synthesized LNPs with tunable elasticity and utilized AFM to quantify the elastic moduli of the LNPs and cells. Our investigation aims to provide insights into how the elastic properties of LNPs and cells influence cellular uptake, tumor permeation, and ultimately, the antitumor efficacy of LNPs in modulated environments.

## 2 Results

### 2.1 Successful Synthesis of Multilayered LNPs with Tunable Elasticity

**Figure 1:**
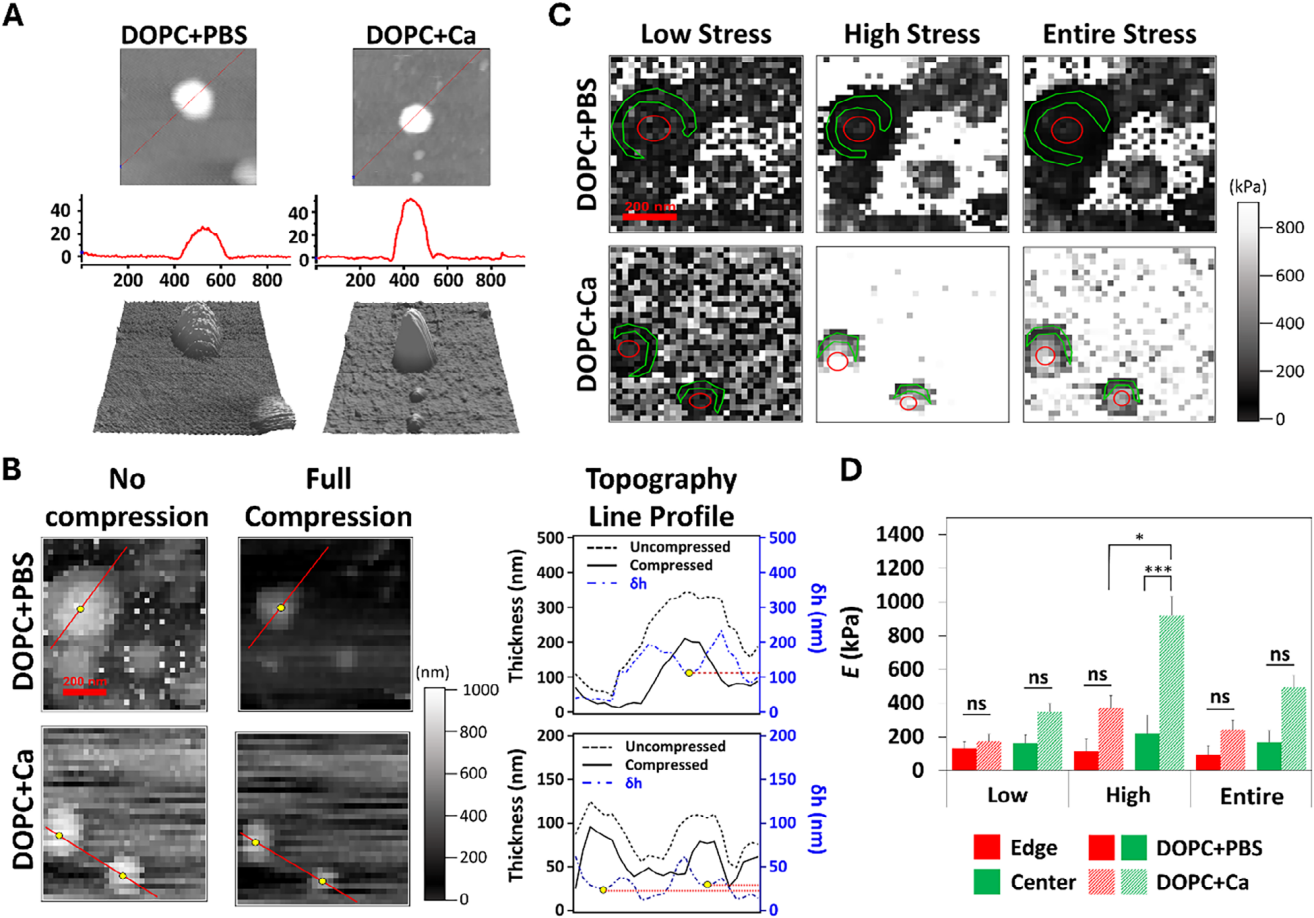
Topographical and mechanical properties of the lipid nanoparticles (LNPs) obtained using atomic force microscopy (AFM). A) Topographical images acquired using AFM tapping mode with line profiles along the red lines. B) Uncompressed and compressed topographies of lipid nanoparticles (LNPs) derived from a 2D force–distance (*f*–*d*) map with line profiles representing the cross‐sections. Each image is 700 × 700 nm with a resolution of 21.875 nm per direction. C) Representative 2D maps of the elastic moduli of LNPs under different stress regimes obtained from a 2D *f*–*d* map. D) Average elastic moduli were measured at the centers and edges of DOPC+PBS and DOPC+Ca LNPs under various stress regimes (*n* = 5 per group). Data are presented as mean ± standard deviation (SD). Statistical significance was determined using a two‐tailed Student's *t*‐test; ^*^ *p* < 0.05, ^***^ *p* < 0.005; ns: non‐significant (*p* > 0.05).

LNPs comprising a lipid bilayer membrane and an internal core of calcium‐crosslinked alginate were synthesized using thin‐film hydration and extrusion methods as described in a previous study.^[^ ^19b^ ^]^ The LNPs were named according to the composition of their internal core: 1,2‐dioleoyl‐sn‐glycero‐3‐phosphocholine (DOPC)+PBS without alginate and DOPC+Ca with alginates cross‐linked with 5 × 10^−3^ m calcium chloride. These LNPs displayed consistent size, polydispersity (PDI), and zeta potential. Dynamic light scattering (DLS) analysis revealed that the average diameters of DOPC+PBS and DOPC+Ca were 158.2 ± 7.4, and 157.4 ± 5.4 nm, respectively (Figure S1A, Supporting Information). In addition, the average PDIs were less than 0.2 for all LNPs, indicating a homogeneous size distribution (Figure S1B, Supporting Information). The zeta potentials for DOPC+PBS, and DOPC+Ca were ‐1.9 ± 4.4 and ‐1.5 ± 4.6 mV, respectively suggesting a proximity to a neutral state with a slightly negative charge distribution (Figure S1C, Supporting Information). The well‐structured spherical shape of the LNPs was confirmed using AFM tapping‐mode imaging (**Figure** **1**A). Despite the consistent size and PDI of all LNPs reported from the DLS data, the line profiles of the AFM images showed a decrease in the height of DOPC+PBS compared to that of DOPC+Ca (Figure 1A), indicating compression by the stress exerted by the AFM probe during imaging. The true topography without compression and the fully compressed topography, obtained by analyzing the *f–d* curves, as described in our previous study, identified the core–shell structure of the LNPs.^[^ ^19b^ ^]^ As shown in Figure 1B, the LNPs in the uncompressed topography display larger and higher coverage than those in the fully compressed topography, revealing the soft shell and hard‐core structures of the LNPs. From the topography line profiles, the differences between the uncompressed and fully compressed topographies were much more exaggerated for DOPC+PBS than that for DOPC+Ca, implying the mechanically softer features of DOPC+PBS. The average elastic moduli of the core and shell of the LNPs were calculated from the regions depicted in red and green, respectively, on the map of the elastic moduli obtained under a constant force of 500 pN (Figure 1C). In the low‐stress regime, the elastic moduli of centers of DOPC+PBS and DOPC+Ca appeared to be different (163 ± 48 and 348 ± 375 kPa, respectively), but no statistical difference was observed. The elastic moduli of the outer edges (129 ± 42 and 173 ± 126 kPa, respectively) also did not differ significantly (Figure 1D, Table S1, Supporting Information). This finding suggests that the mechanical deformation in the low‐stress regime is similar across both the centers and outer edges of the LNPs. However, in the high‐stress regime, the elastic moduli of DOPC+PBS were 216 ± 115 and 115 ± 73 kPa at the center and edge, respectively. For DOPC+Ca, these values were 917 ± 601 and 371 ± 313 kPa, respectively (Figure 1D, Table S1, Supporting Information). These findings indicate that the outer edges, mainly comprising lipid bilayers, are softer than the inner cores, regardless of the chemical composition of the inner cores, as evaluated in the low‐stress regime. However, the findings in the high‐stress regime showed that the inner cores exhibited varying stiffness, with high calcium concentrations leading to increased stiffness due to enhanced cross‐linking of alginate.

Next, we encapsulated doxorubicin (DOX) in the LNPs using the pH gradient method to assess the effects of the LNPs on the antitumor efficacy and pharmacokinetics of DOX. The encapsulation efficiency (EE) and loading capacity (LC) of DOX in the LNPs were 80–90% and 4.27–4.93%, respectively (**Table** **1**). Additionally, the in vitro release profile measured over 9 days at pH 7.4 showed that the cumulative release (CR) at 24 h was 48% and 17% for DOPC+PBS and DOPC+Ca, respectively. Later, the drug release in DOPC+Ca doubled by the endpoint, whereas DOPC+PBS reached 57% release by 108 h with no significant increase thereafter (Figure S1D, Supporting Information). These findings revealed a decrease in CR of DOX in mechanically stiffer LNPs.

**Table 1:** Encapsulation efficiency (EE) and loading capacity (LC) of the lipid nanoparticles (LNPs).

	EE [%]	LC [%]
DOPC+PBS	91.60 ± 3.73	4.93 ± 0.80
DOPC+Ca	84.69 ± 5.69	4.27 ± 0.45

### 2.2 Soft Nature of Malignant Breast Cancer Cells and Protumoral Macrophages

**Figure 2:**
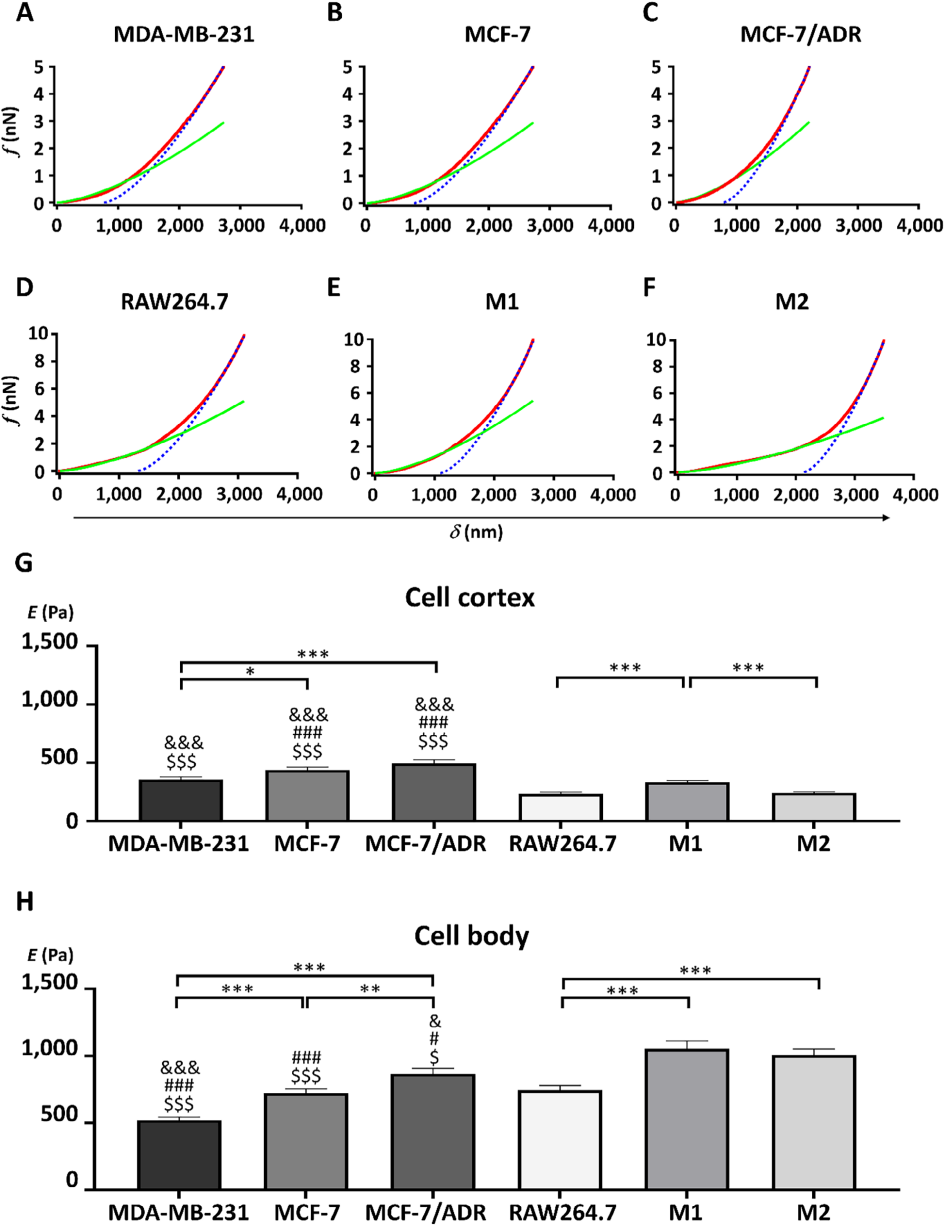
Elasticity of breast cancer cells and macrophages determined from the atomic force microscopy (AFM) indentation experiments. A–F) Representative force–indentation (ƒ–δ) curves from breast cancer cells (A–C) and macrophages (D–F). G,H) Average elasticity of the cell cortex (G) and body (H) for MDA‐MB‐231, MCF‐7, MCF‐7/ADR, RAW264.7, M1, and M2 cells measured at a trigger force of 5 nN for MDA‐MB‐231, MCF‐7, MCF‐7/ADR, RAW264.7, M1, and M2, respectively (*n* = 40 for each cell line). Error bars show standard error of the mean (SEM). Statistical significance was determined using a two‐tailed Student's *t*‐test; ^*^ *p* < 0.05, ^***^ *p* < 0.005; ^&&&^ *p* < 0.005 versus RAW264.7; ^#^ *p* < 0.05, ^###^ *p* < 0.005 versus M1; ^$$$^ *p* < 0.005 versus M2.

To determine the elastic moduli of cells, *f*–*d* curves, which were converted to force–indentation (*f*–*δ*) curves, were obtained from AFM‐indentation experiments using constant forces of 5 and 10 nN for breast cancer cells (**Figure** **2**A–C) and macrophages (Figure 2D–F), respectively. The average elastic moduli of the cell cortex under low‐stress conditions (*f* < 1 nN) were 356 ± 152, 439 ± 162, and 494 ± 209 Pa for MDA‐MB‐231, MCF‐7, and MCF‐7/ADR cells, respectively, indicating the highest mechanical compliance in MDA‐MB‐231 cells (Figure 2G). Under high‐stress conditions (*f* > 4 nN), the elastic moduli of these cells were 523 ± 141, 723 ± 211, and 869 ± 246 Pa, respectively (Figure 2H). These findings suggest that invasive MDA‐MB‐231 cells exhibit higher mechanical compliance, as indicated by their lower elastic moduli, compared to non‐invasive, drug‐sensitive MCF‐7 cells. Conversely, drug‐resistant MCF‐7/ADR cells exhibited increased stiffness, as reflected by their higher elastic moduli.

To explore mechanical interactions among LNPs, cancer cells, and stromal cells, we evaluated the elastic properties of macrophages including RAW264.7 cells and chemically polarized M1 and M2 phenotypes. The successful polarization of macrophages was confirmed by the enhanced expression of inducible nitric oxide synthase (iNOS) and arginase 1 (Arg‐1) in the M1 and M2 phenotypes, respectively (Figure S2, Supporting Information). In contrast to breast cancer cells, macrophages show distinct elastic moduli in both the cell cortex and body. The average elasticities of the cell cortex in RAW264.7, M1, and M2 macrophages measured under low stress (*f* < 2 nN) were 234 ± 98, 333 ± 108, and 242 ± 66 Pa, respectively (Figure 2G). In particular, proinflammatory M1 macrophages displayed higher mechanical stiffness in both the cortex and body than other phenotypes. The elastic moduli of the macrophage cell bodies under high stress (*f* > 8 nN) were 745 ± 215, 1055 ± 369, and 1009 ± 275 Pa for RAW264.7, M1, and M2 cells, respectively (Figure 2H). Nonpolarized macrophages exhibited greater mechanical compliance than their polarized counterparts.

Macrophages generally showed higher elasticity in cell bodies than that in breast cancer cells: RAW264.7 versus MDA‐MB‐231 (*p* < 0.005); M1 versus MDA‐MB‐231 or MCF‐7 (*p* < 0.005); M1 versus MCF‐7/ADR (*p* < 0.05); M2 versus MDA‐MB‐231 (*p* < 0.005) or MCF‐7 (*p* < 0.005) or MCF‐7/ADR (*p* < 0.05) (Figure 2H). As for cell cortices, macrophages were more compliant than all breast cancer cells (*p* < 0.005) (Figure 2G). Nonetheless, a few exceptions were observed; the cell bodies of MCF‐7/ADR were stiffer than those of RAW264.7 cells, and cell cortices of MDA‐MB‐231 cells were not mechanically discernible from those of M1 macrophages. Nevertheless, the major differences in the mechanical properties between breast cancer cells and macrophages likely play a significant role in their interactions with LNPs.

### 2.3 Improved Internalization of Soft LNPs into Soft Cells

**Figure 3:**
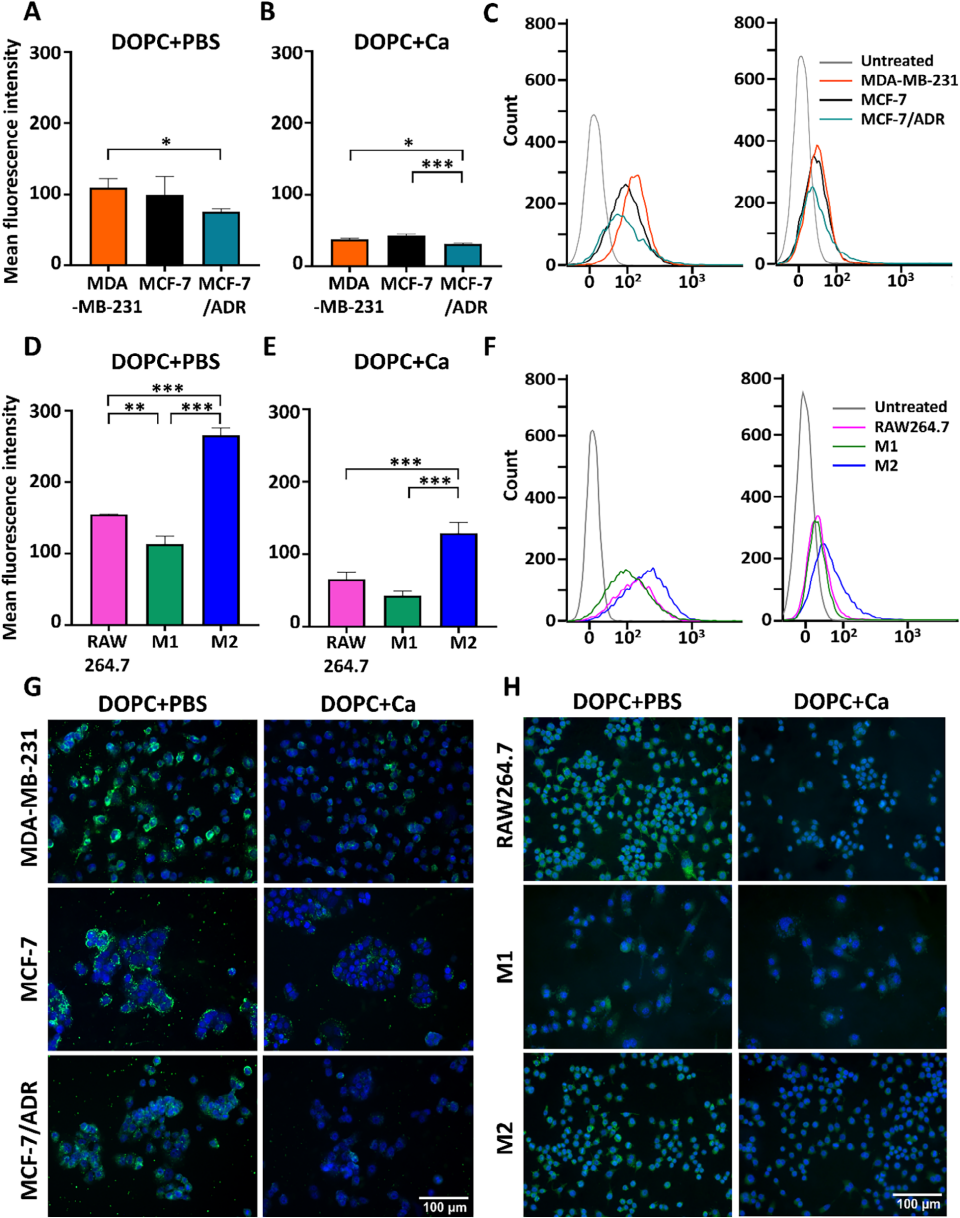
Differences in cellular uptake between low‐elasticity (DOPC+PBS) and high‐elasticity lipid nanoparticles (LNPs) (DOPC+Ca). A,B,D,E) Mean fluorescence intensity (MFI) obtained via flow cytometry representing the internalization rates of LNPs into breast cancer cells (A,B) and macrophages (D,E; *n* = 3 per group). Statistical significance was determined using a two‐tailed Student's *t*‐test. C,F) Representative graphs showing the number of breast cancer cells (C) and macrophages (F) versus fluorescence intensity. G,H) Fluorescence images of LNPs internalized into breast cancer cells (G) and macrophages (H; blue: DAPI, green: LNPs); ^*^ *p* < 0.05, ^**^ *p* < 0.01, ^***^ *p* < 0.005. Error bars show standard error of the mean (SEM).

The fluorescence intensity measured by flow cytometry (**Figure** **3**A–F) indicated the extent of LNP internalization in each cell type. The internalization of soft LNPs (DOPC+PBS) was higher across all cell types than that of stiffer LNPs (DOPC+Ca). Furthermore, soft LNPs exhibited 2.43‐, 2.31‐, and 2.91‐fold higher uptake in MCF‐7/ADR, MCF‐7, and MDA‐MB‐231 cells, respectively, than their stiff counterparts (Figure 3A,B). Similarly, in M2, RAW264.7, and M1 macrophages, we observed 2.05‐, 2.37‐, and 2.60‐fold higher internalization of soft LNPs, respectively (Figure 3D,E). Consistently, the fluorescence intensity of DOPC+PBS was higher than that of DOPC+Ca across all cell types (Figure 3G,H).

Additionally, we found that softer cells internalized more LNPs than stiffer cells did. For instance, DOPC+PBS showed approximately 1.10‐ and 1.45‐fold higher internalization in MDA‐MB‐231 cells than those in MCF‐7 and MCF‐7/ADR cells, respectively (Figure 3A). MDA‐MB‐231 cells exhibited the softest mechanical properties among the investigated breast cancer cell lines (Figure 2G,H). Conversely, the mechanically stiffest MCF‐7/ADR cells showed the lowest uptake of both DOPC+PBS and DOPC+Ca. MDA‐MB‐231 cells exhibited a more significant decrease in the uptake of DOPC+Ca than DOPC+PBS (Figure 3B). Fluorescence microscopy images also demonstrated consistent results, with a gradual decrease in fluorescence intensity from MDA‐MB‐231 to MCF‐7 to MCF‐7/ADR cells (Figure 3G), indicating reduced LNP internalization in stiffer breast cancer cells. This trend was observed for both DOPC+PBS and DOPC+Ca.

Similar to breast cancer cells, LNP uptake in macrophages is strongly dependent on their mechanical compliance. Soft M2 macrophages displayed higher internalization of both DOPC+PBS and DOPC+Ca than that of stiffer M1 macrophages (Figure 3D and E). Specifically, the cellular uptake of DOPC+PBS in M2 macrophages was approximately 1.71‐ and 2.35‐fold higher than that in RAW264.7 and M1 macrophages, respectively (Figure 3D). Furthermore, M2 macrophages exhibited 1.97‐ and 2.98‐fold higher uptake of DOPC+Ca than RAW264.7 and M1 cells, respectively (Figure 3E). Fluorescence images confirmed the enhanced internalization of both LNP types in M2 macrophages compared to that in M1 macrophages (Figure 3H). Furthermore, RAW264.7 cells also showed high LNP uptake similar to that of M2 macrophages in fluorescence images (Figure 3H), although flow cytometry showed lower uptake in RAW264.7 than that in M2 macrophages (Figure 3D,E). These results indicated that chemical treatments used for macrophage polarization may have increased the elastic moduli of M1 and M2 phenotypes compared to RAW264.7 cells (Figure 2H), thus affecting LNP uptake (Figure 3H). Overall, the findings showed that both types of LNPs exhibited increased uptake in softer cells.

### 2.4 Enhanced Internalization of LNPs into Breast Cancer Cells in the Presence of Protumoral Macrophages

**Figure 4:**
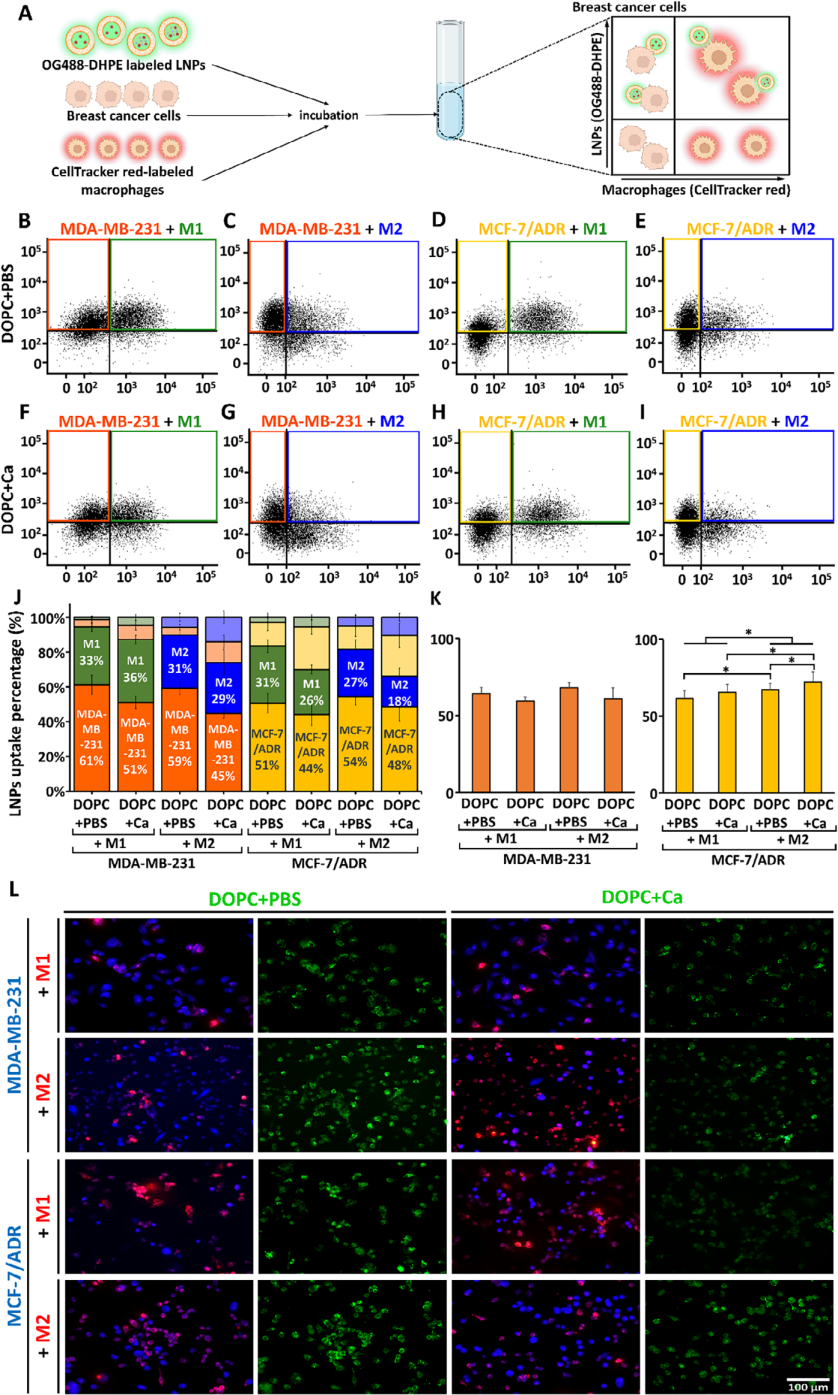
Effect of interaction between breast cancer cells and macrophages on lipid nanoparticle (LNP) internalization. A) Schematic illustration of the measurement of LNP internalization in a coculture system of breast cancer cells and macrophages. Macrophages labeled with Cell Tracker Red CMTPX dye were visually discernible from breast cancer cells; thus, the amount of OG488‐DHPE‐labeled LNPs internalized into each cell type was measured using flow cytometry (FACS). B–I) Representative flow cytometry dot plots showing selective uptake of soft (B–E) and stiff (F–I) LNPs by different cell types in the coculture system. Colors represent MDA‐MB‐231, MCF‐7/ADR, M1, and M2 cells. J) Cumulative percentiles of fluorescence intensity generated by soft and stiff LNPs internalized into each cell type in each coculture system. Error bars show standard error of the mean (SEM). K) Relative uptake of soft and stiff LNPs into breast cancer cells compared with the total number of cells internalized with LNPs in each coculture system. Error bars represent standard error of the mean (SEM) (*n* = 5 per group). Statistical significance was determined using a two‐tailed Student's *t*‐test; ^*^ *p* < 0.05. L) Representative fluorescence images showing soft and stiff LNPs (green) internalized by breast cancer cells (blue) and macrophages (red).

To investigate whether the presence of macrophages with different polarization states affects the internalization of LNPs into breast cancer cells, we conducted flow cytometry using OG488‐DHPE‐labeled LNPs in a coculture system consisting of breast cancer cells and macrophages labeled with CellTracker Red CMTPX dye, as illustrated in **Figure** **4**A. Representative dot plots and quadrant images from FACS analysis showed the amount of LNPs internalized into each cell type within the coculture system (Figure 4B–I). The upper left quadrants represent LNPs internalized into breast cancer cells, whereas the upper right quadrants display LNPs internalized into macrophages. Breast cancer cells and macrophages without LNPs are shown in the lower left and right quadrants, respectively. The average LNP uptake for each cell type is shown in the cumulative plots in Figure 4J. Similar to the results obtained from the single‐cell culture system, soft LNPs demonstrated more efficient internalization into cells than stiff LNPs, regardless of the cell type combination in each coculture system.

We observed significant increases in the uptake of both soft and stiff LNPs into MDA‐MB‐231 cells in the presence of M1 and M2 macrophages compared to the uptake into MCF‐7/ADR cells. This finding is consistent with those from the single cell‐culture system, showing that LNPs are more efficiently internalized into mechanically softer cells. To minimize any ambiguity due to improper gating in FACS analysis, we calculated the relative uptake of LNPs into breast cancer cells among all LNP‐positive cells, as shown in Figure 4K. The findings showed increased internalization of LNPs into MCF‐7/ADR cells in the presence of M2 macrophages compared to that of M1 macrophages, irrespective of the stiffness of the LNPs. We speculated that this difference may be due to the protumorigenic nature of M2 macrophages and the malignancy of MCF‐7/ADR cells. In contrast, no significant change was observed in MDA‐MB‐231 cells, which maintained high malignancy under all conditions. Similar observations were obtained for the fluorescence images shown in Figure 4L. These images confirmed the increased internalization of soft LNPs compared to that of stiff LNPs in both coculture systems. The coculture of MDA‐MB‐231 cells and M2 macrophages represented a combination of mechanically compliant cells, whereas the coculture of MCF‐7/ADR cells and M1 macrophages involved stiffer cells. A significant increase in LNP internalization was observed in the coculture of MDA‐MB‐231 cells and M2 macrophages compared to that in the coculture of MCF‐7/ADR cells and M1 macrophages.

### 2.5 Energy‐Efficient Internalization Pathways of LNPs into Breast Cancer Compared with Macrophage

**Figure 5:**
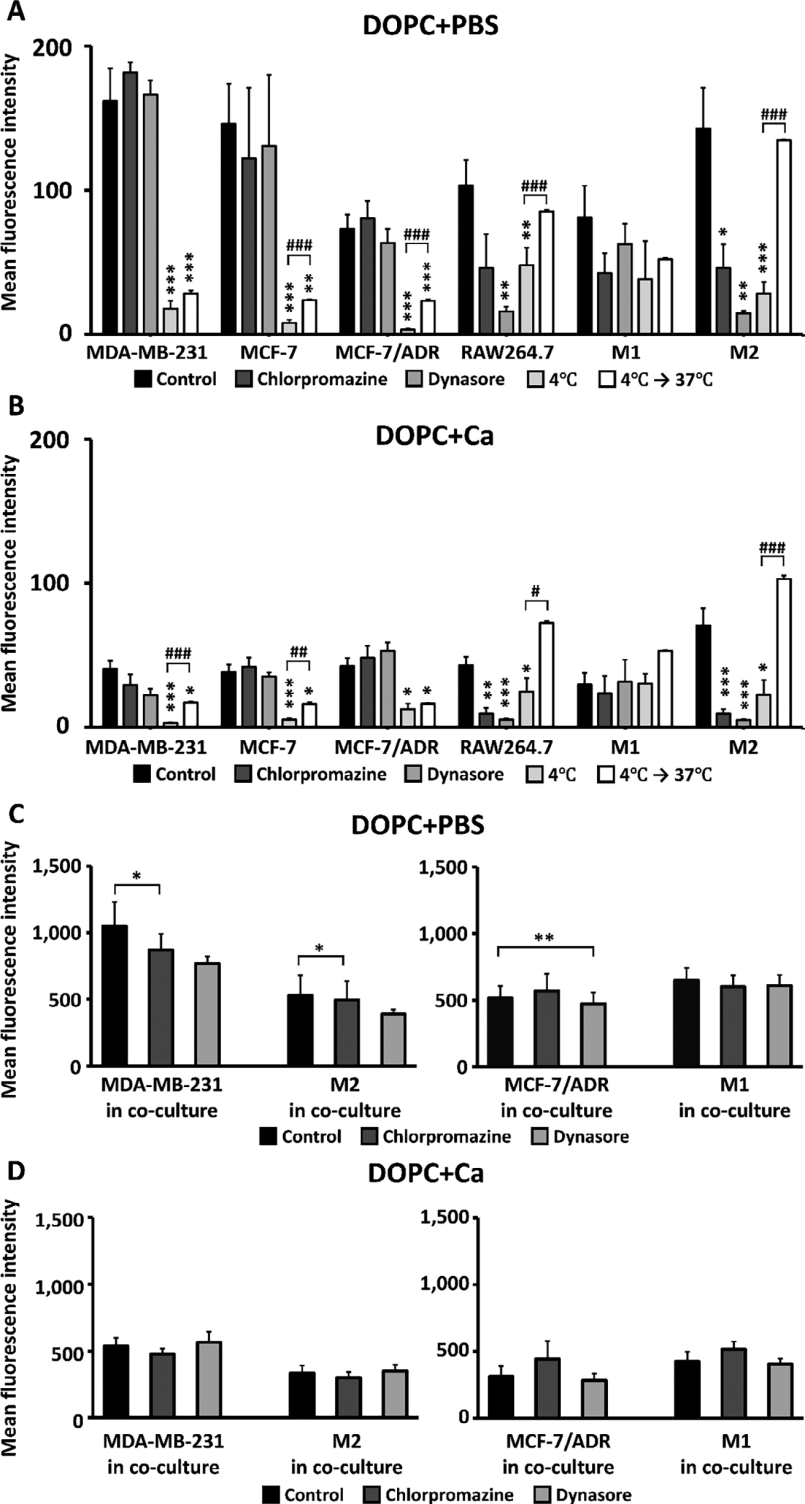
Endocytosis pathways of soft and stiff lipid nanoparticles (LNPs) in breast cancer cells and macrophages. A,B) Changes in the cellular uptake of soft (A) and stiff (B) LNPs upon treatment with chlorpromazine (clathrin‐mediated endocytosis inhibitor), dynasore (clathrin/caveolae‐mediated endocytosis inhibitor) and 4 °C (fusion inhibition; *n* = 3 per group). C,D) Identification of endocytosis pathways of soft (C) and stiff (D) LNPs in a coculture system when inhibiting endocytosis (*n* = 4 per group). Statistical significance was determined using a two‐tailed Student's *t*‐test; ^*^ *p* < 0.05, ^**^ *p* < 0.01, ^***^ *p* < 0.005 versus Control; ^#^ *p* < 0.05, ^##^ *p* < 0.01, ^###^ *p* < 0.005 4 °C versus 37 °C after 4 °C; Error bars show standard error of the mean (SEM).

Next, we investigated the differences in endocytic pathways due to the modulated elasticity of cells and LNPs by treating cells with various endocytosis inhibitors, including chlorpromazine (a clathrin‐mediated endocytosis inhibitor) and dynasore (a clathrin/caveolae‐mediated endocytosis inhibitor). We also observed fusion events of the LNPs by conducting experiments under low‐temperature conditions. First, we observed a substantial decrease in the internalization of LNPs into all breast cancer cells at 4 °C, but no change was detected upon addition of endocytosis inhibitors, indicating that LNP uptake by breast cancer cells primarily occurs via energy‐efficient fusion (**Figure** **5**A,B). The reduced uptake of LNPs into breast cancer cells at 4 °C was mostly recovered by the successive incubation at physiological temperature, proving that LNPs enter breast cancer cells via fusion. Although soft LNPs showed enhanced internalization compared with those of their stiffer counterparts, no difference in the internalization pathways was observed between soft and stiff LNPs in breast cancer cells.

However, macrophages utilized both fusion and endocytosis pathways, and the specifics of their internalization pathways varied according to polarization state and LNP type. The internalization of both soft and stiff LNPs into M1 macrophages was not significantly affected by any condition that inhibited the internalization pathways. Unlike M1 macrophages, M2 macrophages exhibited a reduced uptake of both types of LNPs following treatment with endocytosis inhibitors and low‐temperature experiments, indicating active endocytosis and fusion mechanism. In RAW264.7 cells, the internalization of stiff LNPs was reduced by both chlorpromazine and dynasore, whereas that of soft LNPs was inhibited by dynasore and low temperature. Except for M1 macrophages, the reduced uptake of LNPs was recovered at M2 and RAW264.7 cells when incubating them at 37 °C after low‐temperature incubation, which indicates that fusion is one of the major pathways used by macrophages to internalize LNPs. These results suggest that in RAW264.7 cells, stiff LNPs primarily undergo clathrin‐mediated endocytosis, whereas soft LNPs utilize caveolae‐mediated endocytosis and fusion.

Furthermore, we explored cell‐specific endocytic pathways based on the elasticity of LNPs in a coculture system of breast cancer cells and macrophages. When endocytic trafficking was inhibited, no distinct endocytic pathways were observed in breast cancer cells under coculture conditions (Figure 5C,D). M2 macrophages displayed a significant decrease in the internalization of both types of LNPs following treatment with chlorpromazine and dynasore, indicating active endocytosis. This result provides further evidence for the enhanced internalization of LNPs into breast cancer cells compared with M2 macrophages in coculture systems. Clathrin‐ and caveolae‐mediated endocytosis necessitates membrane deformation and the formation of coated pits, requiring cells to expend more time and energy to internalize LNPs. These findings indicate that M2 macrophages consume more energy than breast cancer cells, leading to selective internalization of LNPs into breast cancer cells in coculture systems. Similar to M2 macrophages, we observed a decrease in the internalization of soft LNPs into M1 macrophages following treatment with dynasore but not chlorpromazine. However, this inhibition was not observed for the stiff LNPs. We did not conduct experiments at low temperatures because of the clear results observed in single‐cell culture systems (Figure 5A,B). Taken together, these findings suggest that the internalization pathways of LNPs primarily depend on cell type. LNPs enter breast cancer cells via an energy‐efficient fusion pathway. However, macrophages internalize soft LNPs through clathrin‐ and caveolae‐mediated endocytosis and stiff LNPs through endocytosis and fusion.

### 2.6 Enhanced Permeation of Soft LNPs into Spheroids Made of Soft Breast Cancer Cells

**Figure 6:**
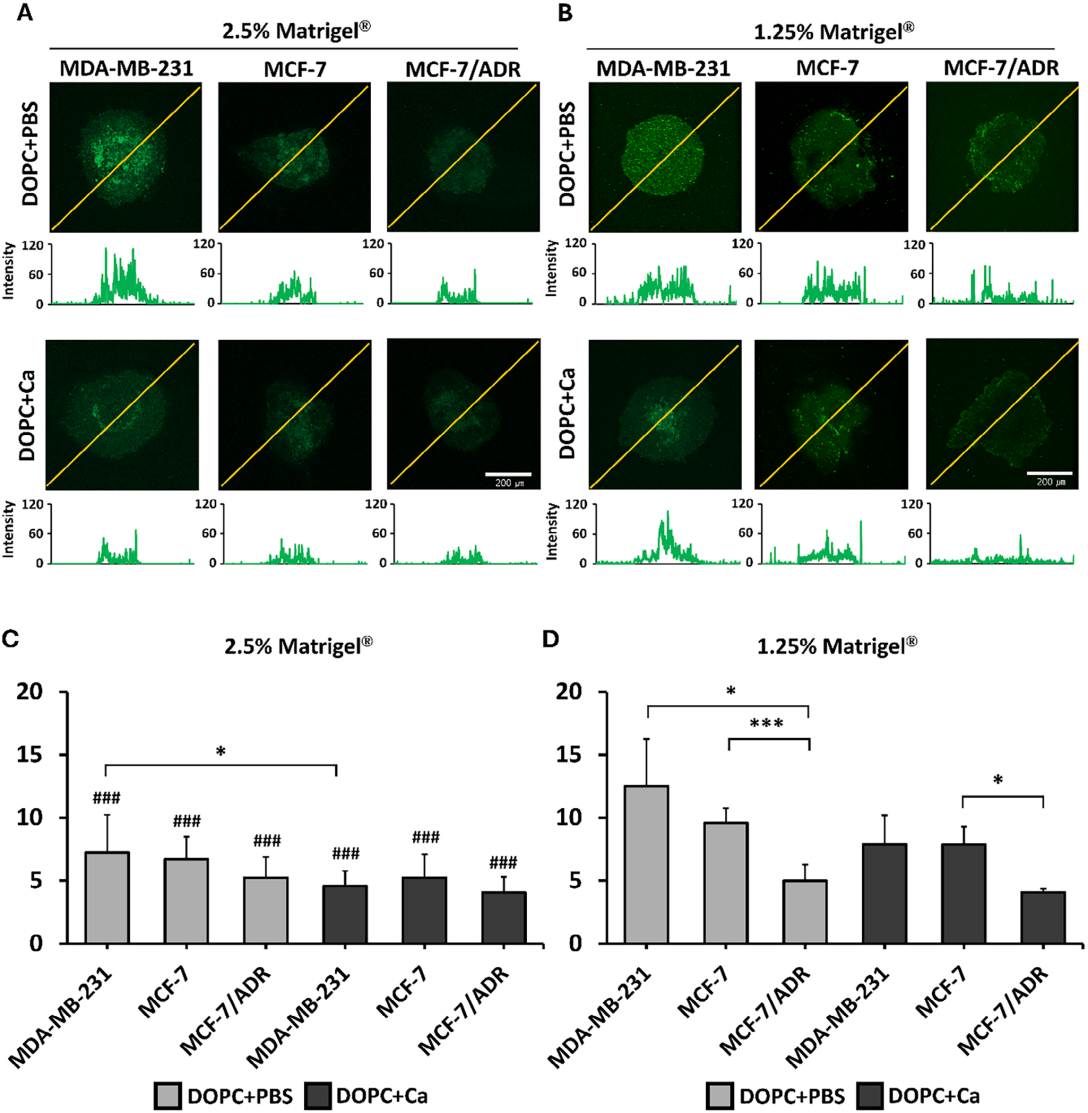
Permeation of lipid nanoparticles (LNPs) labeled with green fluorescence into breast cancer spheroids with different Matrigel concentrations. Confocal images with line profiles along the yellow lines for A) 2.5% and B) 1.25% of Matrigel. Average fluorescence intensity at different Matrigel concentrations – C) 2.5% and D) 1.25% (*n* = 3 per group). Statistical significance was determined using a two‐tailed Student's test. Data are presented as mean ± standard deviation (SD); ^*^ *p* < 0.05 and ^***^ *p* < 0.005 for the indicated comparison; ^###^ *p* < 0.005 versus spheroid with 1.25% Matrigel.

We investigated whether the mechanical properties of the LNPs affect their permeation into 3D spheroids. The exemplary confocal images taken from breast cancer spheroids treated with fluorescently labeled LNPs are presented in **Figure** **6**A,B, along with line profiles visualizing their penetration depth and distribution. The average fluorescent intensity was calculated to quantify the permeation of LNPs into each group of spheroids (Figure 6C,D). Our findings showed that the soft DOPC+PBS LNPs exhibited considerably stronger permeation into spheroids than those of their stiffer counterparts (Figure 6). The enhanced permeation of soft LNPs was more pronounced in spheroids composed of mechanically compliant MDA‐MB‐231 cells. Conversely, LNPs showed minimal penetration into spheroids formed from stiffer MCF‐7/ADR cells. This variation in LNP uptake between the different types of spheroids was more significant in spheroids containing 1.25% Matrigel than in those with 2.5% Matrigel. Furthermore, spheroids formed with 1.25% Matrigel (Figure 6B,D) demonstrated higher LNP uptake than those formed with 2.5% Matrigel (Figure 6A,C). The reduced entanglement in 1.25% Matrigel might facilitate the easier penetration of LNPs into spheroids. These findings suggest that not only cellular elasticity but also the mechanical stiffness of the surrounding tumor microenvironment influences the permeation of LNPs into tumors.

### 2.7 Rapid Penetration of Soft LNPs and Prolonged Retention of Stiff LNPs into Tumors

**Figure 7:**
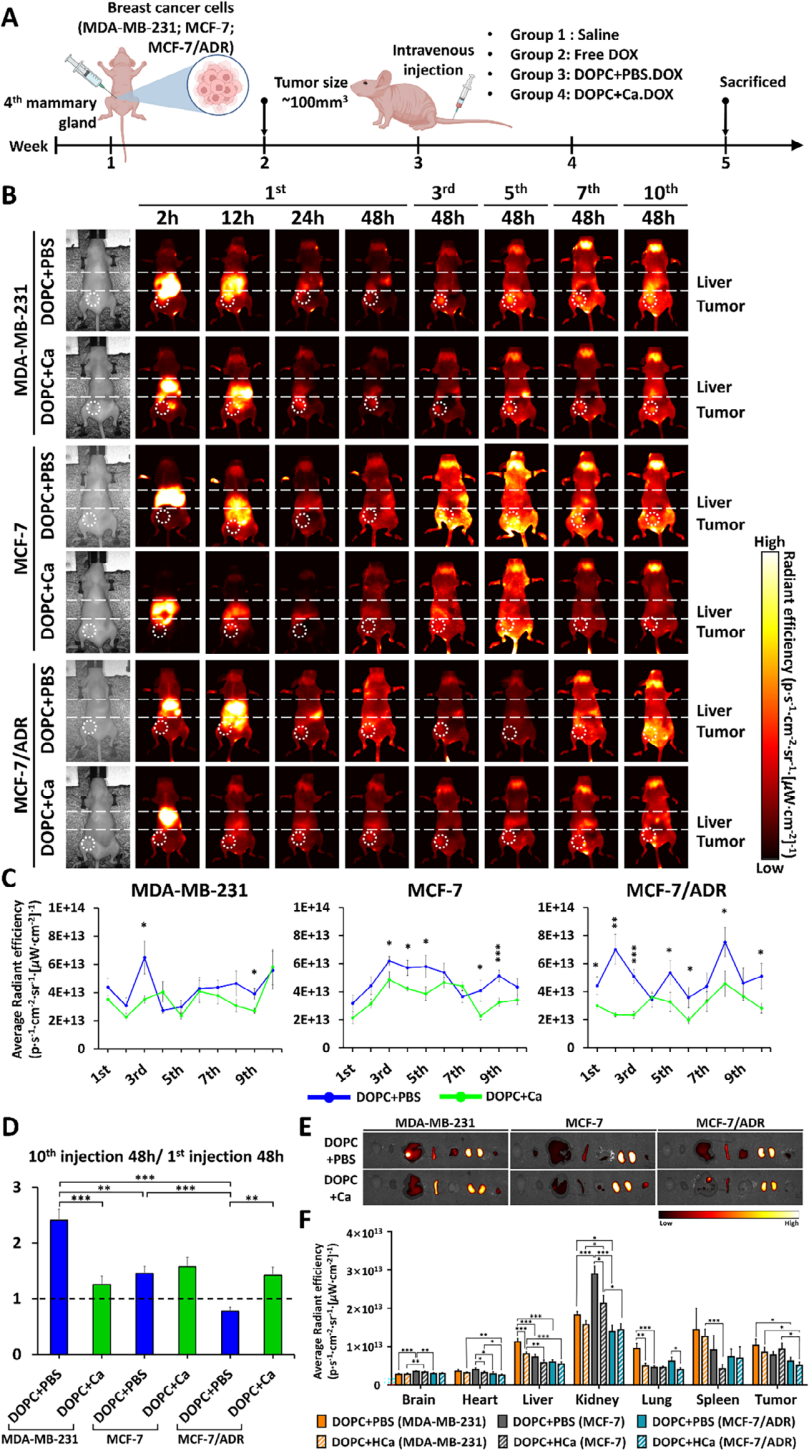
Biodistribution and antitumor efficacy of Cy5.5‐labeled DOPC+PBS.DOX and DOPC+Ca.DOX in orthotopic breast cancer models (*n* = 5). A) Schematic representation of the treatment regimen and tumor establishment in BALB/c nude mice. Ten intravenous injections were administered every 48 h after the tumor volume reached 100 mm^3^. B) In vivo fluorescence images showing the biodistribution of Cy5.5‐labeled DOPC+PBS and DOPC+Ca administered intravenously to mice with breast cancer. C) Fluorescence intensities of soft and stiff lipid nanoparticles (LNPs) observed in mice 2 h after each administration. D) Quantification of LNP accumulation by calculating the ratio of fluorescence intensities at 48 h after the 10^th^ injection relative to the 1^st^ injection. E) Fluorescence images of LNP‐treated tumors and major organs (brain, heart, liver, spleen, lung, and kidney) harvested from sacrificed mice at the experimental endpoint. F) Average radiant efficiencies from tumors and major organs harvested from LNP‐treated mice. Statistical significance for C,D,F) was determined using a two‐tailed Student's *t*‐test; ^*^ *p* < 0.05, ^**^ *p* < 0.01, ^***^ *p* < 0.005. Error bars represent standard deviation (SD).

Next, we evaluated the in vivo distribution and tumor accumulation of LNPs with varying mechanical deformability. LNPs encapsulated with DOX—DOPC+PBS.DOX (soft LNPs) and DOPC+Ca.DOX (stiff LNPs) were labeled with Cy5.5 and administered according to the schedule shown in **Figure** **7**A. Representative images of orthotopic breast cancer‐bearing mice that received multiple injections of LNPs are shown in Figure 7B. Two hours after the first injection of both LNP types, the fluorescence signal from Cy5.5‐labeled LNPs began to appear at the tumor sites in almost every mouse with orthotopic breast cancer. At 12 h post‐injection, LNPs started to accumulate at the tumor sites, although some mice with MCF‐7/ADR‐derived tumors did not display a strong fluorescence signal at the tumor sites before 12 h after the first injection. Up to 12 h, soft LNPs at the tumor sites exhibited stronger fluorescence intensity than stiff LNPs in all mice. The fluorescence intensity data at 24 h showed that the LNPs began to be excreted from the animal bodies, including the tumor sites. Furthermore, at 2 h after each administration, soft LNPs showed stronger fluorescence intensities than stiff LNPs in all mice, indicating elevated permeation of soft LNPs into the tumor sites (Figure 7C).

Subsequently, the accumulation of LNPs at the tumor sites was quantified by calculating the ratio of fluorescence intensities at 48 h after the 10^th^ injection to those after the first injection (Figure 7D). Consistent with the permeation observed in Figure 7C, soft LNPs showed 1.92 times higher accumulation than stiff LNPs in tumors established from MDA‐MB‐231 cells (*p* < 0.05). However, tumors established from MCF‐7/ADR cells showed the opposite results; stiff LNPs accumulated 82% more than soft LNPs (*p* < 0.01). No significant difference in this ratio was observed in MCF‐7‐derived tumors. We also confirmed that the accumulation of soft LNPs decreased as soft breast cancer cells adapted to form tumors, whereas stiff LNPs did not display this correlation.

Fluorescent images of the tumors harvested from the sacrificed animals (Figure 7E; Figure S3, Supporting Information) also confirmed that soft LNPs accumulated 66% (*p* < 0.05) and 36% more in tumors derived from MDA‐MB‐231 and MCF‐7 cells than in those derived from MCF‐7/ADR cells (Figure 7F). Similarly, the fluorescence intensity of stiff LNPs in tumors derived from MDA‐MB‐231 cells showed a 67% increase compared with that in tumors derived from MCF‐7/ADR cells (*p* < 0.05). The accumulation of stiff LNPs in tumors established from MCF‐7 cells was 1.82 times higher than that in tumors established from MCF‐7/ADR cells (*p* < 0.05). Moreover, stiff LNPs displayed 19% more fluorescence intensity in tumors derived from MCF‐7 cells than soft LNPs, indicating a higher accumulation of stiff LNPs.

**Figure 8:**
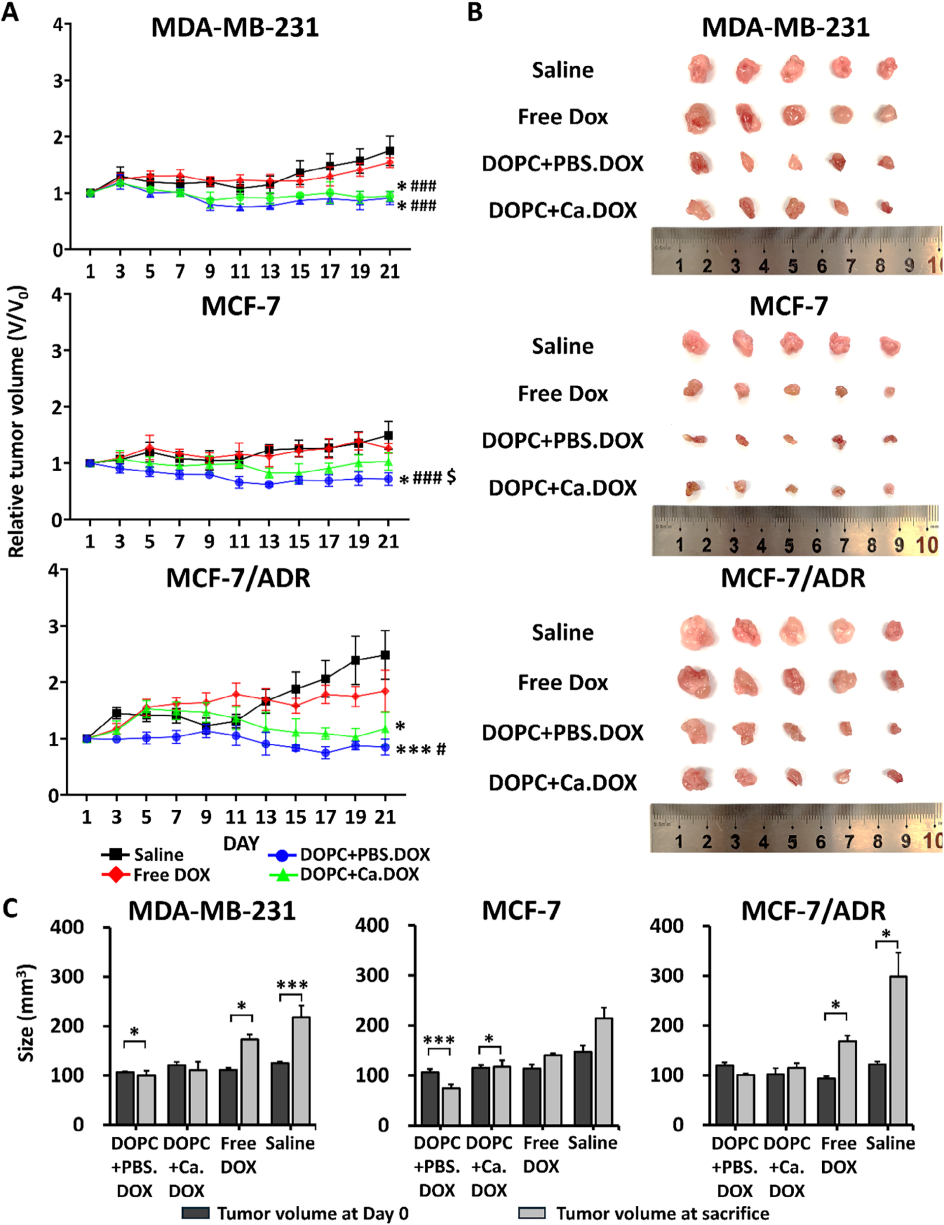
Antitumor efficacy of DOPC+PBS.DOX and DOPC+Ca.DOX in orthotopic breast cancer models (*n* = 5). A) Relative tumor volume compared with the initial tumor volume in each treatment group over time; ^*^ *p* < 0.05, ^***^ *p* < 0.005 versus saline; ^#^ *p* < 0.05, ^###^ *p* < 0.005 versus free DOX; ^$^ *p* < 0.05 versus DOPC+PBS.DOX. B) Photograph of resected tumors from breast tumor‐bearing mice treated with saline, free DOX, DOPC+PBS.DOX, or DOPC+Ca.DOX. C) Tumor size measured in tumor‐bearing mice before the treatment and after resection at the experimental endpoint; ^*^ *p* < 0.05, ^***^ *p* < 0.005. Statistical significance for A,C) was determined using a two‐tailed Student's *t*‐test. Error bars represent standard deviation (SD).

During treatment, the soft LNP‐treated mice bearing tumors established from all the observed breast cancer cells displayed decreases in the relative tumor volume based on the tumor size on the date of the first injection (**Figure** **8**A). None of the mice displayed any change in body weight during the observation period (Figure S4, Supporting Information). Mice bearing tumors derived from MDA‐MB‐231 cells showed a −5.72% and −8.87% decrease in relative tumor volume after treatment with soft and stiff LNPs, respectively. However, MCF‐7 cell‐derived tumor‐bearing mice showed −27.97% and 2.93% changes in the relative tumor volume after treatment with soft and stiff LNPs, respectively. We also found a 63% greater reduction in the relative tumor volume after treatment with soft LNPs than with stiff LNPs (*p* < 0.05). The MCF‐7/ADR cell‐derived mouse model showed similar results to the MCF‐7 cell‐derived mouse model; changes in relative tumor volume were −14.95% and 17.66% with treatment with soft and stiff LNPs, respectively.

As shown in Figure 8B,C, the size of tumors harvested from MDA‐MB‐231 cell‐derived tumor‐bearing mice treated with soft LNPs was decreased (*p* < 0.05). Additionally, the sizes of MCF‐7‐derived (*p* < 0.005) and MCF‐7/ADR‐derived tumors also decreased after treatment with soft and stiff (*p* < 0.005 and *p* < 0.05 for MCF‐7 only, respectively). Although the changes in MCF‐7/ADR‐derived tumors were not statistically significant, they still hold meaningful efficacy, considering the significant increases in tumor size in the saline‐and free DOX‐treated groups.

## 3 Discussion

The physicochemical properties of LNPs play a significant role in their pharmaceutical performance, influencing systemic circulation and tissue permeation. Recently, the mechanical stiffness of LNPs has been investigated in relation to their targeting ability and cellular uptake.^[^ ^6^, ^29^ ^]^ However, these studies yielded inconsistent conclusions due to the complex nature of interactions between LNPs and cells.^[^ ^30^ ^]^ Understanding these interactions is further complicated by the dynamic relationship between cancer cells and their surrounding microenvironments, which can modulate both mechanical and chemical properties.^[^ ^31^ ^]^ To address this complexity, we explored the triangular mechanical interplay between LNPs, cancer cells, and the tumor microenvironment. This interplay plays a crucial role in influencing the cellular uptake, tumor accumulation of LNPs, and the malignant progression of cancer cells.

While consistent efforts have led to the identification of optimal size and charge for LNPs to effectively deliver payloads to tumor sites, optimizing the stiffness of LNPs remains a challenge due to difficulties in both modulating and accurately measuring their mechanical properties. To address this issue, we synthesized LNPs with an alginate core surrounded by a lipid bilayer. By maintaining consistent chemical compositions for the outer lipid membrane, we modulated Young's moduli of the alginate cores by varying the degree of cross‐linking through different calcium concentrations. DLS data confirmed no significant differences in size or zeta potential among the investigated LNPs. Additionally, AFM experiments revealed calcium concentration‐dependent increases in the Young's modulus of the formulated LNPs. The physical properties, including Young's moduli of our LNPs, were consistent with previously reported values for nanolipogels (NLGs) with similar chemical compositions.^[^ ^27^ ^]^ Sun et al. also evaluated the effect of the elasticity of liposomes on the treatment of acute lung injury by varying Young's moduli of phosphatidylserine‐containing liposome from 2 to 100 kPa, which lies within the order of magnitude of Young's moduli of our LNPs.^[^ ^18^ ^]^ Furthermore, LNPs, including liposomes utilized for drug delivery in clinical applications, are mainly composed of phosphatidylcholine lipids, similar to our LNPs. The lipid composition of Doxil, the first FDA‐approved liposomal doxorubicin, is also composed of phosphatidylcholine lipids with cholesterol and PEGylation. Although increases in elastic moduli were expected owing to the existence of cholesterol and PEGylation, we believe that the elastic moduli of our LNPs lie within the range of elastic moduli of commercially available nanocarriers. Moreover, the obtained maps of Young's modulus under varying stress regimes showed that changes in Young's modulus were attributable to the stiff alginate cores, while the outer membrane of the LNPs exhibited consistent mechanical behavior. Generally, drug carriers, including LNPs, are designed to provide structural integrity, enhance payload protection, and regulate payload release kinetics.^[^ ^32^ ^]^ Consistently, in this study, the stiff LNPs (DOPC+Ca) exhibited slow drug release over an extended period compared to the soft LNPs (DOPC+PBS).

The diverse chemical compositions of NPs inherently lead to a wide range of Young's moduli, complicating the understanding of how NP elasticity affects cellular uptake. Inconsistent findings may also stem from variations in the types of target cells and NPs used. Therefore, optimizing NP stiffness for cellular internalization is crucial. Studies on silica NPs, with Young's moduli ranging from 560 kPa to 4.7 GPa, consistently show that stiffer silica NPs are internalized more efficiently than softer ones.^[^ ^4^ ^]^ This difference is attributed to variations in deformation and endocytosis pathways during cell‐NP interactions.^[^ ^33^ ^]^ However, this correlation does not hold for silica NPs modified with cell membrane coatings or hyaluronic acid,^[^ ^3^, ^34^ ^]^ indicating that surface modifications can alter uptake patterns.

In the case of polymeric hydrogel NPs, reports on the relationship between NP elasticity and cellular uptake are conflicting.^[^ ^14^, ^17^, ^26^, ^35^ ^]^ For example, Liu et al. found that softer hydrogel NPs made of poly(2‐hydroxyethyl methacrylate) exhibited faster and greater internalization into HepG2 cells than those of their stiffer counterparts.^[^ ^26^ ^]^ Chen et al. observed similar results under dynamic flow conditions,^[^ ^35^ ^]^ where enhanced adhesion between soft NPs and cells was identified as a major contributing factor. Conversely, Anselmo et al. reported that softer hydrogel NPs composed of polyethylene glycol showed reduced cellular uptake in immune, endothelial, and breast cancer cells.^[^ ^17^ ^]^ These softer hydrogel NPs demonstrated prolonged circulation and enhanced tumor‐targeting abilities compared with those of stiffer ones, suggesting that softer NPs may improve pharmaceutical performance. Focusing on lipid‐based NPs, Guo et al. demonstrated that soft NLGs (Young's moduli (*E*) < 1.6 MPa) had greater internalization into both neoplastic and non‐neoplastic cells compared to those of stiffer NLGs (*E* > 13.8 Mpa).^[^ ^27^ ^]^ Consistent with these findings, our data revealed that soft LNPs were internalized more efficiently into breast cancer cells and macrophages than stiffer LNPs. This trend was also observed in the internalization of LNPs into breast cancer spheroids and tumors in an orthotopic breast cancer model.

Over the past decades, a key finding from AFM‐based biomechanical studies is that cancer cells are mechanically more compliant than normal cells, and cancer progression significantly alters the mechanical compliance of cancer cells.^[^ ^7^, ^36^ ^]^ During internalization, both LNPs and cells undergo mutual deformation. In this study, we observed substantial increases in LNP internalization in softer cells. Our AFM‐indentation experiments confirmed that all breast cancer cells studied were softer than RAW264.7 macrophages, regardless of whether they were M1 or M2 polarized. MDA‐MB‐231 breast cancer cells exhibited the highest mechanical compliance and demonstrated unparalleled LNP uptake compared with other cell types, indicative of their malignant phenotype. These observations remained consistent across various experimental models, including single cells, spheroids, and animal models. LNPs exhibited deeper and more extensive penetration in spheroids formed by soft MDA‐MB‐231 cells. In the animal model, LNPs rapidly permeated the tumors formed by soft breast cancer cells. These findings align with previous studies showing that softer cancer cells have elevated levels of clathrin and caveolin‐1 proteins, which are key regulators of endocytic pathways.^[^ ^37^ ^]^ Additionally, lower plasma membrane tension in softer cells promotes dynamic deformation during clathrin‐mediated endocytosis,^[^ ^38^ ^]^ thereby enhancing cellular uptake. Our data also revealed that softer M2 macrophages exhibited greater LNP internalization than that of stiffer M1 macrophages, which suggests that chemical activation‐induced changes in the elastic moduli of macrophages influence LNP uptake. For example, Patel et al. documented a 1.7‐ to 2.7‐fold increase in elastic moduli with IFN‐γ and LPS treatments, respectively.^[^ ^39^ ^]^ These findings suggest that increased mechanical compliance in target cells facilitates LNP internalization.

Our findings from the coculture system suggest a competitive dynamic between cancer cells and macrophages in the internalization of LNPs. When cocultured with M2 macrophages, stiff MCF‐7/ADR cells internalized more LNPs than those cocultured with M1 macrophages. In the tumor microenvironment, the interaction between tumor‐associated macrophages (TAMs) and cancer cells plays a significant role in cancer progression and treatment. TAMs, a key component of immune cells infiltrating tumors, exhibit different activation states and functions.^[^ ^40^ ^]^ One of the key characteristics of TAMs is their ability to adopt either protumor (M2‐like) or anti‐tumor (M1‐like) properties. M2‐like TAMs are often associated with promoting tumor progression by supporting processes such as angiogenesis, immunosuppression, and tissue remodeling. In contrast, M1‐like TAMs typically exert cytotoxic effects on tumor cells and support anti‐tumor immune responses.^[^ ^41^ ^]^ Given this complex dynamics, the presence of TAMs can impact LNP internalization, depending on their polarization. M2‐like TAMs secrete various cytokines and growth factors contributing to immunosuppression and tissue remodeling.^[^ ^42^ ^]^ These factors reorganize the tumor microenvironment by modulating cancer cell surface receptor expression and endocytic pathways. Notably, the transforming growth factor‐β (TGF‐β) signaling pathway regulates clathrin‐mediated endocytosis and macropinocytosis, both critical for NP uptake.^[^ ^43^ ^]^ Additionally, interleukin‐10 may enhance LNP internalization by altering cytoplasmic organization and cell membrane fluidity.^[^ ^44^ ^]^ Furthermore, C‐C motif chemokine ligand 18 (CCL18), a cytokine linked to tumor progression, promotes the malignant transformation of cancer cells and may also increase NP uptake.^[^ ^45^ ^]^ A recent study by Brill–Karniely et al. suggested that more aggressive cancer cells exhibit an elevated capability to uptake NPs.^[^ ^46^ ^]^ Additionally, M2‐like TAMs may facilitate NP uptake by cancer cells via phagocytosis or by altering the expression of cell surface receptors or transporters on cancer cells, thereby creating a conducive environment for NP uptake. It is plausible that the aggressiveness of cancer cells, facilitated by interactions with M2‐like TAMs and other components of the tumor microenvironment, could enhance their capability to uptake NPs. Consistent with this, we observed that breast cancer cells cocultured with M2 macrophages showed increased internalization of LNPs compared to those cocultured with M1 macrophages. This supports the tendency of soft cells and cancer cells in the presence of M2 macrophages to internalize more LNPs.

From the perspective of drug delivery, the diversification of internalization pathways and the energy consumption involved are crucial for the successful delivery of LNPs into target cells. Multiple endocytic pathways are available for LNPs to enter target cells.^[^ ^47^ ^]^ We explored the internalization pathways used by LNPs to enter breast cancer cells and macrophages by treating cells with chlorpromazine and dynasore. Chlorpromazine, a cationic amphiphilic drug, disrupts the formation of clathrin‐coated pits by translocating clathrin and its adaptor proteins from the plasma membrane to intracellular vesicles. Dynasore, a reversible inhibitor of dynamin, inhibits membrane disassembly during clathrin‐mediated endocytosis and also blocks clathrin‐independent, dynamin‐dependent pathways, including caveolae‐mediated endocytosis.^[^ ^48^ ^]^ Additionally, cells were incubated at 4 °C to prevent LNP fusion with the plasma membrane. As a result, our findings revealed that LNPs entered breast cancer cells primarily through direct fusion, an energy‐efficient pathway. In contrast, macrophages internalized LNPs predominantly via clathrin‐ and caveolae‐dependent endocytosis, a process that requires relatively higher energy expenditure. Consequently, LNPs were internalized more efficiently by cancer cells than by macrophages in the coculture system. The existence of macrophages in the coculture system created a more dynamic microenvironment for cancer cells. Signaling molecules, such as cytokines, along with physical interactions originating from macrophages, enhanced the reliance on endocytic pathways in breast cancer cells. This, in turn, increased their sensitivity to chlorpromazine and dynasore treatments. The diversified internalization pathways in breast cancer cells likely contributed to the preferential uptake of LNPs by cancer cells compared with macrophages in the coculture system. Previous studies have offered different views on the internalization pathways of NPs in cancer cells and macrophages.^[^ ^4^, ^27^ ^]^ For instance, Hui et al. reported that macrophages primarily utilized phagocytosis and macropinocytosis, while ovarian cancer cells depended on a clathrin‐ and caveolae‐independent pathway for NP internalization.^[^ ^4b^ ^]^ Conversely, Guo et al. showed that soft NLGs predominantly underwent fusion, with less reliance on endocytosis, while stiffer NLGs favored energy‐dependent endocytosis.^[^ ^27^ ^]^ Although further investigations are warranted to fully elucidate the mechanisms underlying LNP internalization in different conditions, our findings align with other observations, particularly regarding the diversity of internalization pathways. Understanding the mechanisms of LNP internalization enables the development of therapies tailored to exploit specific cellular properties. For example, therapeutic strategies could be designed to preferentially target tumor cells via fusion‐mediated pathways while minimizing off‐target effects in macrophages.

In the TME, the ECM plays a crucial role in tumor cell mobility, immune cell infiltration, and the delivery of NP‐based drug cargo. The density and structural organization of the ECM govern the mechanical stiffness of tumor tissue, directly influencing the diffusion and intracellular uptake of NP. In particular, ECM stiffness increases due to the accumulation and cross‐linking of structural proteins such as collagen, leading to the formation of a denser and more rigid structure as the tumor progresses.^[^ ^49^ ^]^ He et al. reported that a denser ECM network restricts NP diffusion, whereas a more loosely arranged or degraded ECM facilitates greater NP penetration.^[^ ^12^ ^]^ Consistent with this notion, we observed enhanced LNP uptake and penetration as the concentration of Matrigel decreased. Matrigel is a biomimetic ECM containing laminin, collagen IV, and various ECM proteins.^[^ ^50^ ^]^ While its higher concentration results in a dense ECM and an intensified physical barrier, lower concentrations contribute to increases in the ECM mesh size, promoting LNP diffusion and allowing NPs to reach spheroids more efficiently. These results suggest that the density and structural organization of the ECM can significantly impact LNP delivery and that the physical characteristics of the ECM are key factors in controlling LNP delivery efficiency.

The findings of this study highlight the substantial impact of LNP elasticity on their pharmacokinetic behavior and therapeutic efficacy. Soft LNPs rapidly entered tumor sites but quickly escaped after repeated administration, whereas stiff LNPs showed more gradual initial penetration and remained in the tumor for a longer duration. Moreover, soft LNPs exhibited extended accumulation at tumor sites in mouse models established with soft breast cancer cells (MDA‐MB‐231). However, in models established with stiff breast cancer cells (MCF‐7/ADR), stiff LNPs accumulated more in the tumor sites. The enhanced permeability of soft LNPs encapsulated with DOX exhibited increased tumor suppression, consistent with the findings of Guo et al.^[^ ^27^ ^]^ Although the prolonged retention of stiff LNPs at tumor sites may contribute to antitumor efficacy, the ability of soft LNPs to rapidly permeate the tumor plays a critical role in tumor suppression. This rapid penetration could be partly attributed to their effective escape from the reticuloendothelial system, leading to decreased uptake by immune cells, including macrophages, as suggested by Desai et al.^[^ ^51^ ^]^ However, we observed that the relative difference in tumor suppression between soft and stiff LNPs was less than 15%. Furthermore, in the MCF‐7 model, stiff LNPs reduced the tumor size by 2.84% more than soft LNPs. These findings indicate that despite their reduced cell uptake, the prolonged retention of stiff LNPs at the tumor site allows them to act as sustained‐release drug reservoirs, which may enhance their therapeutic effects over time.

**Figure 9:**
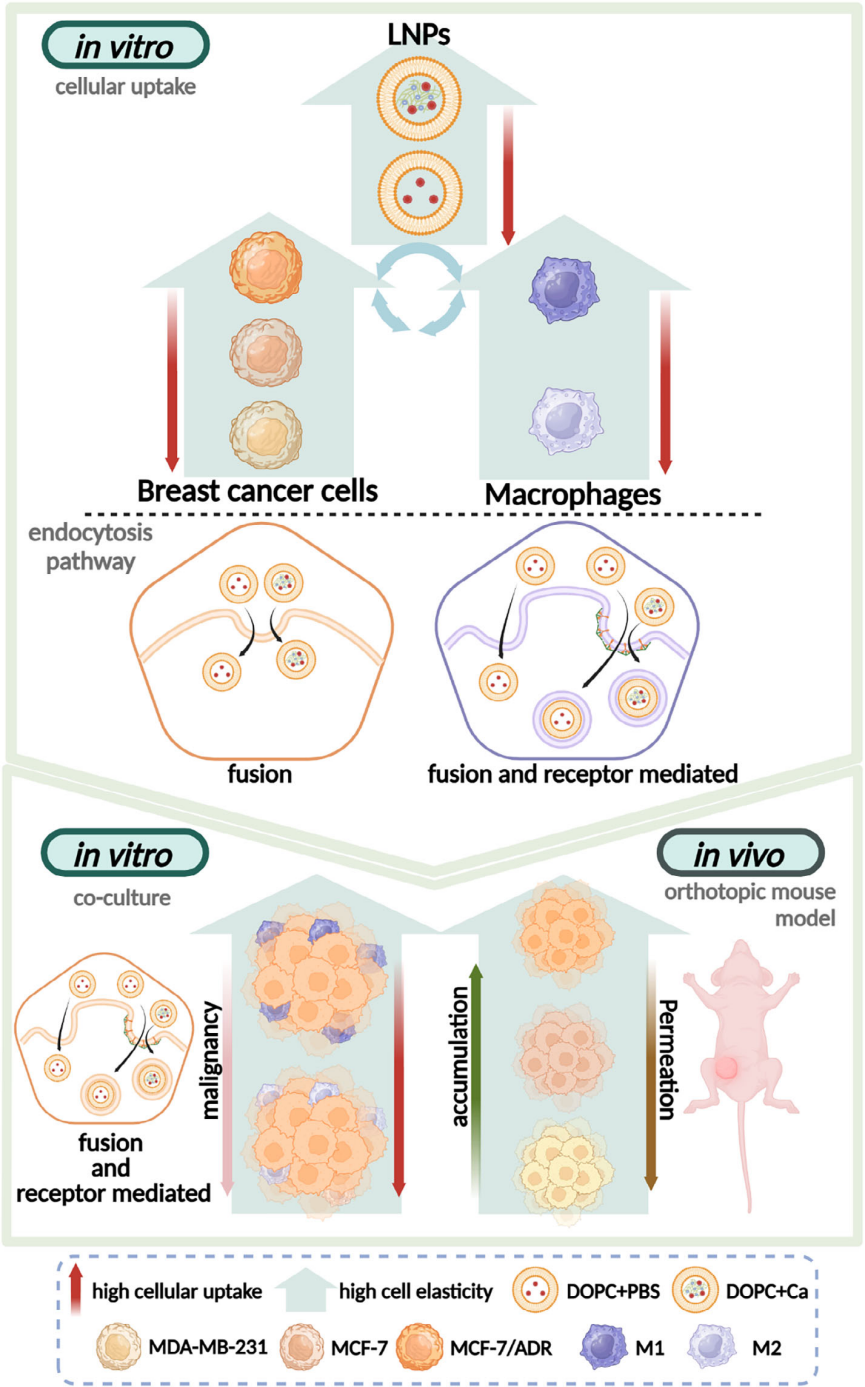
Schematic illustration depicting the cellular uptake and internalization mechanisms influenced by the mechanical properties of lipid nanoparticles (LNPs), breast cancer cells, and macrophages.

As shown in **Figure** **9**, our study demonstrates that the mechanical properties of LNPs are crucial for optimizing pharmacokinetic behavior and improving chemotherapeutic efficacy. Specifically, soft LNPs exhibit rapid cellular uptake, resulting in more effective tumor suppression. In contrast, stiff LNPs show prolonged retention at tumor sites in mouse models established with MCF‐7 and MCF‐7/ADR cells, contributing to enhanced antitumor activity. In addition to the stiffness of LNPs, the mechanical properties of cells also influence the rate of LNP internalization; our study revealed that softer cells exhibit enhanced LNP internalization. Moreover, interactions between cancer cells and other cells within the tumor microenvironment—particularly protumoral macrophages—play a critical role in LNP internalization. Breast cancer cells in the presence of M2 macrophages, which are associated with tumor progression, showed increased LNP uptake. The softness of cancer cells and the presence of protumoral macrophages are closely linked to the malignancy and invasiveness of tumors. Thus, the enhanced internalization of LNPs by softer cancer cells, and by cells within protumoral microenvironments, represents a phenomenon intricately tied to cancer malignancy. Overall, our findings underscore that the triple mechanical interaction among the properties of LNPs, cancer cells, and the tumor microenvironment is a critical factor in the determination of the pharmaceutical behavior and therapeutic efficacy of LNP‐based drug delivery systems.

## 4 Conclusion

In this study, we demonstrated that the mechanical interplay between LNPs and the tumor microenvironment significantly influences both LNP internalization into cancer cells and their penetration into tumor sites. LNPs were fabricated by controlling the cross‐linking of alginate cores wrapped in a lipid bilayer, resulting in particles with tailored elastic moduli. Our results showed that softer LNPs were more effectively internalized by all observed cell types, with higher efficiency noted in softer cancer cells (MDA‐MB‐231) and softer macrophages (M2). In coculture with macrophages, cancer cells accounted for more than 50% of the total cellular uptake. This is likely due to cancer cells utilizing an energy‐efficient fusion pathway, whereas macrophages predominantly employ receptor‐mediated endocytosis. Furthermore, the presence of M2 macrophages increased the internalization of both soft and stiff LNPs into stiffer breast cancer cells (MCF‐7/ADR), suggesting that the mechanical compliance of cancer cells correlates with their malignant and invasive characteristics. Given that M2 macrophages contribute to the establishment of protumoral microenvironments that enhance malignancy, the observed increase in LNP uptake by soft cancer cells and the enhanced internalization in the presence of M2 macrophages can be explained by the elevated malignancy within the tumor microenvironment. In orthotopic mouse models of breast cancer established using three different cell lines, soft LNPs demonstrated enhanced permeation into tumor sites. After multiple administrations, the accumulation of soft LNPs was higher in mouse models established from soft MDA‐MB‐231 cells, while stiff LNPs showed increased accumulation in models established from stiff MCF‐7/ADR cells. Moreover, our LNP formulation encapsulated with DOX exhibited higher tumoricidal efficacy compared to free DOX. Specifically, soft LNPs showed more potent antitumor effects across all animal models, likely due to their enhanced permeation into tumor sites. Together, our findings underscore the critical role of the mechanical properties of both LNPs and the tumor microenvironment, including cancer cells, in effective LNP‐based cancer therapy. These insights highlight the importance of mechanical properties in the development of tailored anticancer strategies.

## 5 Experimental Section

### Preparation of LNPs

LNPs comprising a lipid bilayer and an aqueous core were synthesized using thin‐film hydration and extrusion methods, following the procedure described in a previous study.^[^ ^19b^ ^]^ Briefly, 25.44 µmol 1,2‐dioleoyl‐sn‐glycero‐3‐phosphocholine (DOPC; Sigma‐Aldrich, St. Louis, MD, USA) was dissolved in chloroform. The mixture was then evaporated using a rotary evaporator to form a thin film, which was subsequently dried overnight in a vacuum desiccator to completely remove the residual solvent. The dried thin film was hydrated with PBS (DOPC+PBS) or sodium alginate solution at pH 7.4, and the suspension was subjected to five freeze–thaw cycles using liquid nitrogen and a heating block to obtain unilamellar vesicles. The suspension was extruded nine times through a 200 nm filter (Avanti Polar Lipids, Alabaster, AL, USA) to obtain uniform LNPs of less than 200 nm. Free sodium alginate was removed by dialysis using 10 K molecular weight cut‐off SnakeSkin dialysis tubing (Sigma‐Aldrich). Subsequently, 5 × 10^−3^ m CaCl_2_ (1:1 w/w) was added as an ionic cross‐linker to control the degree of cross‐linking of the alginate core and thus obtain LNPs (DOPC+Ca) with varying elastic moduli. To synthesize fluorescently labeled LNPs, 0.4 mol% Oregon Green‐488 1,2‐dihexadecanoyl‐sn‐glycero‐3‐phosphoethanolamine (OG488‐DHPE; Invitrogen, Carlsbad, CA, USA) was added during the initial formation of the lipid thin film.

### DLS Analysis

The hydrodynamic size, PDI, and zeta potential of the prepared LNPs were evaluated using DLS (Brookhaven Instruments Co., Holtsville, NY, USA). All measurements were performed using 5 × 10^−3^ m LNPs in distilled water.

### Topography and Mechanical Analysis Using AFM

All AFM measurements were performed using a commercial AFM instrument (MFP3D; Asylum Research, Santa Barbara, CA, USA) following the experimental and analytical procedures described in a previous study.^[^ ^19b^ ^]^ For the AFM measurements of the LNPs, the LNPs were doped onto an amino‐salinized mica surface. AFM topographic images of the LNPs were obtained in the tapping mode using a triangular silicon nitride cantilever (Bruker, microlever, 20 kHz, 0.07 N m^−1^) in an aqueous medium at 15–25 °C with 700 × 700 nm^2^ at 256 × 256‐pixel resolution and a 0.5 Hz scan rate. To evaluate the elastic moduli of the LNPs, the AFM spectroscopy was performed generating *f*–*d* curves in a two‐dimensional array over a single LNP, referred to as “force volume data.” The linearity of the force and scanner movements was ensured by obtaining the *f*–*d* curves on hard substrates. AFM‐based indentation experiments were performed to mechanically characterize the cells. Briefly, cells were cultured on a glass slide at a density of 1×10^4^ cells. An AFM probe attached to polystyrene spheres with a diameter of 4.5 µm was used to minimize the stress applied to the cells by the probe during the AFM measurements. To maintain the physiological activity of the observed cells, AFM measurements were performed within 3 h of removing cells from the cell culture incubator. The *f*–*d* curves were obtained with a trigger force of 5–20 pN at the center of the cells by selecting measurement points using an optical microscope where the AFM was installed. Similar to the AFM spectroscopy of the LNPs, scanner linearity was confirmed by obtaining *f*–*d* curves for a hard substrate. The uncompressed topography of the LNPs was delineated from the force volume data by determining the contact points from the approaching *f*–*d* curves, as described in a previous study.^[^ ^19b^ ^]^ From the force volume data, a map of the elastic moduli for a single LNP was delineated using hyperbolic fitting of a modified Hertz model, considering the asymptotic behavior of the *f*–*d* curves. The details of the analysis have been explained in a previous study.^[^ ^19b^ ^]^ Similarly, the elastic moduli of the cells were determined from the approaching *f*–*d* curves obtained from the AFM‐indentation experiments.^[^ ^19a^ ^]^

### EE and LC

DOX (Sigma‐Aldrich) was loaded into the LNPs using the pH gradient method. Briefly, LNPs were formed using a solution containing 250 × 10^−3^ m ammonium sulfate (pH 5.4), and any free ammonium sulfate solution was removed using a PD‐10 desalting column (Cytiva, Marlborough, MA, USA). DOX (5 mg mL^−1^) was then encapsulated into the LNPs via a pH gradient at a pH of 7.4, with a drug‐to‐lipid ratio of 1:20 (w/w). The mixture was stirred at 60 °C for 1 h, and the unloaded DOX was removed using a PD‐10 column. The amount of DOX encapsulated in the LNP was confirmed using a UV‐visible spectrophotometer (UV‐1800; Shimadzu Corporation, Kyoto, Japan) at 480 nm, following lysis with 1% Triton X‐100. Drug EE and LC were calculated using the following equations:

(1) $$\mathrm{EE}\left(\%\right)=\frac{\mathrm{Encapsulated}\;\mathrm{amount}\;\mathrm{of}\;\mathrm{DOX}}{\mathrm{Total}\;\mathrm{amount}\;\mathrm{of}\;\mathrm{DOX}}\times 100$$

(2) $$\mathrm{LC}\left(\%\right)=\frac{\mathrm{Total}\;\mathrm{amount}\;\mathrm{of}\;\mathrm{DOX}-\mathrm{Free}\;\mathrm{Dox}}{\mathrm{Total}\;\mathrm{amount}\;\mathrm{of}\;\mathrm{DOX}\;\mathrm{loaded}\;\mathrm{in}\;\mathrm{liposomes}}\times 100$$

### Drug Release

Drug release was monitored using dialysis and was assessed at different time intervals (2, 4, 8, and 12 h, and every 12 h up to 214 h) using a 10 kDa dialysis tube containing 1 mL suspension of DOX‐loaded LNPs suspended in 9 mL PBS (pH 7.4). At each time point, 1 mL of the suspension was withdrawn and an equal volume of fresh PBS (pH 7.4) was added to maintain the volume. Aqueous suspensions of DOX‐loaded LNPs were analyzed at 480 nm using a UV‐visible spectrophotometer. Drug release was calculated using cumulative calculations.

### Cell Culture and Macrophage Polarization

The breast cancer cell line, MCF‐7/ADR, was cultured in Roswell Park Memorial Institute (RPMI) 1640 medium containing l‐glutamine (Hyclone, Marlborough, MA, USA) supplemented with 10% fetal bovine serum (FBS) and 1% penicillin‐streptomycin (Gibco, Rockville, MD, USA). MCF‐7 and MDA‐MB‐231 cell lines were obtained from the Korean Cell Line Bank (KCLB, Seoul, South Korea). For MCF‐7 and MDA‐MB‐231 cells, RPMI 1640 medium containing l‐glutamine supplemented with 10% FBS and 1% penicillin‐streptomycin was used. MCF‐7 cells were additionally supplemented with sodium pyruvate, whereas MDA‐MB‐231 cells were treated with sodium pyruvate and non‐essential amino acids (NEAA) (Gibco). All cells were cultured in a cell incubator under 5% CO_2_ and 37 °C.

Murine macrophages (RAW264.7) were grown in RPMI1640 containing l‐glutamine, supplemented with 10% FBS and 1% penicillin‐streptomycin, in a humidified atmosphere at 37 °C with 5% CO_2_. For M1 macrophage polarization, RAW264.7 were treated with LPS (10 ng mL^−1^) and murine recombinant IFN‐γ (10 ng mL^−1^; Peprotech, Rocky Hill, NJ, USA) for 24 h. To induce M2 macrophages, RAW264.7 were treated with murine recombinant IL‐4 (20 ng mL^−1^; Peprotech).

### Western Blot Assay

Proteins were extracted from RAW264.7 macrophages, as well as M1 and M2 polarized macrophages, using RIPA buffer supplemented with protease and phosphate inhibitors. The protein concentration in each sample was quantified to be 50–60 µg using the BCA assay. Electrophoresis was performed for approximately 2 h at 60 V and 100 V using an 8–12% polyacrylamide gel. Proteins were separated by gel electrophoresis and transferred to polyvinylidene fluoride (PVDF) membranes (Bio‐Rad, Hercules, CA, USA). To minimize nonspecific reactions, the membrane was blocked with 5% bovine serum albumin (BSA), at 15–25 °C, for 1 h. The membrane was then incubated overnight at 4 °C with primary antibodies against GAPDH, Arg‐1, and iNOS primary antibodies (Cell Signaling Technology, Danvers, MA, USA; at 1:1000). Subsequently, the membranes were washed and incubated with an anti‐rabbit horseradish peroxidase‐conjugated secondary antibody (Cell Signaling Technology; 1:5000) for 1.5 h. Finally, ECL solution (GE Healthcare, Chicago, IL, USA) was added for 5 min, and protein expression levels were detected using LAS4000 (GE Healthcare). The expression levels were quantified using ImageJ (National Institutes of Health, Bethesda, MD, USA).

### Immunofluorescence Assay

Cells (5 × 10^5^) were cultured on a cover glass. After 24 h, the medium was removed and the cells were fixed using a 4% paraformaldehyde solution (Biosesang, Seongnam, Korea) for 10 min. The fixed cells were permeabilized with 0.1% Triton X‐100 and reacted at 4 °C for 10 min and then blocked with 5% BSA solution containing 0.05% Tween‐20 for 1 h. The cells were then incubated with primary antibodies against Arg‐1 and iNOS (Cell Signaling Technology) diluted at 1:150 and 1:200, respectively, overnight at 4 °C. Following washing, the cells were treated with Alexa Fluor 644 secondary antibody (Invitrogen; 1:800) and incubated at 15–25 °C for 2 h. Cell nuclei were stained and fixed using ProLong Gold containing DAPI (Invitrogen). Fluorescence images were obtained using an AX10 fluorescence microscope (Carl Zeiss, Oberkochen, Germany).

### Flow Cytometry

Flow cytometry was performed to determine differences in the cellular uptake of LNPs. Each cell line was cultured in 5% CO_2_ at 37 °C with 2 × 10^5^ cells in a 6‐well plate. After 24 h, the medium was removed, and the cells were treated with 100 µL mL^−1^ of OG488‐DHPE‐conjugated LNP for 4 h. To analyze the differences in cellular uptake of the LNPs in cocultures, staining was performed with CellTracker RED CMTPX (Invitrogen) for 30 min before treating the cells with fluorescently labeled LNPs. The cells were then collected by washing three times with PBS and treating them with trypsin. The collected cells were diluted in 1 mL PBS and analyzed using flow cytometry. Fluorescence from OG488‐DHPE was detected using the fluorescein isothiocyanate (FITC) channel, with 10 000 cells gated for each sample. Flow cytometric analysis was performed using the BD FACSFlow system (BD Biosciences, San Jose, CA, USA).

### Endocytosis Inhibitor Treatment

To determine the endocytic pathways involved in LNP uptake, cells were treated with chlorpromazine and dynasore (Sigma‐Aldrich) to inhibit the clathrin and clathrin/caveolin pathways, respectively. Breast cancer cells and macrophages were cultured for 24 h and treated with 20 × 10^−6^ m chlorpromazine or 80 × 10^−6^ m dynasore at 37 °C for 1 h. Cells pretreated with the inhibitors were subsequently incubated with fluorescently labeled LNPs at 37 °C for 4 h. Cellular uptake of LNPs was assessed using FACS. Additionally, cell membrane fusion with LNPs was investigated using thermal deactivation. Cells were pre‐cooled at 4 °C for 1 h without inhibitors, incubated with fluorescently labeled LNPs at 4 °C for 4 h, and cellular uptake was measured using FACS. These results were compared with those of control groups that were not treated with inhibitors or exposed to LNPs at 4 °C.

### Spheroid Model Confocal Microscopy

Each well of a 96‐well plate was coated with 1.5% agarose before adding 5000 cells per well for spheroid formation. After 2 days, spheroids were grown in media containing 1.25% or 2.5% Matrigel (Corning Inc., Corning, NY, USA) per 100 µL for 3 days. After 5 days, spheroid morphology was observed using a microscope. OG488‐DHPE‐conjugated LNPs were added to each well at a concentration of 150 µL mL^−1^ and incubated for 18 h. The medium was then removed and the spheroids were washed and fixed with 4% paraformaldehyde for 10 min. After washing three times, spheroid absorption of the LNPs was observed using an LSM700 confocal laser‐scanning microscope (Carl Zeiss).

### Biodistribution of LNPs in Breast Cancer Orthotopic Mice

Animals were cared for in accordance with the National Institutes of Health's Guide for the Care and Use of Laboratory Animals. All animal experiment protocols were approved by the Institutional Animal Care and Use Committee (IACUC) of Keimyung University (IACUC number: KM2022‐009, approved on 2022.08.19). Female BALB/c nude mice (5 weeks old) were purchased from Hyochang Science (Daegu, Korea) and housed in microisolator cages on an individually ventilated cage rack maintained with a 12‐h light/dark cycle with free access to an autoclaved standard rodent diet. To establish the tumor model, MCF‐7, MCF‐7/ADR, and MDA‐MB‐231 cells (1 × 10^6^ cells/100 µL Matrigel) were injected into the 4^th^ mammary gland of the BALB/c nude mice. When tumor volumes reached approximately 100 mm^3^, Cy5.5‐labeled (I) DOPC+PBS and (II) DOPC+Ca were injected intravenously (*n* = 5 per group). The injections were repeated every 48 h for a total of 10 injections. The distribution of Cy5.5 fluorescence was assessed at 2, 12, 24, and 48 h after each injection using the VISQUE IN VIVO Elite imaging system (Vieworks, Anyang, Korea). Tumors and organs were excised and imaged.

### In Vivo Antitumor Efficacy

Tumors were established using the method described above. When the tumor volume reached 100 mm^3^, the mice were randomly divided into five groups within the same cell group: G1, Saline; G2, Free DOX; and G3, DOPC + PBS. Dox; G4, DOPC+Ca.Dox. Each group consisted of five animals. Tumor size was measured every 2 days, and the volume (*V*) was calculated using the following equation:

(3) $$\mathrm{Volume}\;\left(V\right)=0.5\times A\times B^{2}$$

where *A* is the longest diameter (mm) and *B* is the shortest diameter (mm). Each group received 100 µL of the designated treatment intravenously every alternate day for 21 days. On day 21, the mice were sacrificed and the tumors were excised and photographed.

### Statistical Analysis

Statistical analysis was performed using Microsoft Excel 2016 (Microsoft Corp., Redmond, WA, USA). Data were assessed for normality and outliers before analysis. Results are presented as mean ± standard error of the mean (SEM). Unless otherwise stated, results are presented as mean ± standard deviation (SD). Sample sizes for each analysis are indicated in the figure legends. A two‐tailed Student's *t*‐test was used to determine the significance between the two groups. A *p*‐value less than 0.05 was considered statistically significant.

## Conflict of Interest

The authors declare no conflict of interest.

## Author Contributions

E.L. and L.N.D. contributed equally to this study. E.L., L.N.D., J.C., H.K., and L.B. performed the experiments, developed the methodology, and conducted the investigation and formal analysis. E.L., L.N.D., and S.P. wrote the manuscript. S.P. was responsible for the review, editing, project supervision, funding acquisition, and conceptualization. The manuscript was written with the contributions of all authors. All the authors approved the final version of the manuscript.

## Supporting information



Supporting Information

## Data Availability

The data that support the findings of this study are available from the corresponding author upon reasonable request.
