# Supplementary material for: Elasticity‐Driven Nanomechanical Interaction to Improve the Targeting Ability of Lipid Nanoparticles in the Malignant Tumor Microenvironment
Source: Adv Sci (Weinh). 2025 Mar 27;12(26):2502073. doi: 10.1002/advs.202502073 (PMC12245016; doi:10.1002/advs.202502073)
Supplement: Supplementary file 1 — Supporting Information [file ADVS-12-2502073-s001.docx]

Supporting Information

Elasticity-Driven Nanomechanical Interaction to Improve the Targeting Ability of Lipid Nanoparticles in the Malignant Tumor Microenvironment

Eunhee Lee, Loi Nguyen Dang, Jinsol Choi, Haesoo Kim, Lyndon Bastatas, Soyeun Park*

**Table S1**. Statistical analysis of elastic moduli (E) of lipid nanoparticles (LNPs) measured by AFM. E is presented as mean ± standard deviation. Student’s *t*-test was applied for comparison between groups, and the resulting p-values are indicated.

|  |  | Low (kPa) | High (kPa) | Entire (kPa) |
| --- | --- | --- | --- | --- |
| DOPC+PBS | E at edge | 129 ± 42 | 115 ± 73 | 91 ± 57 |
|  | E at center | 163 ± 48 | 216 ± 115 | 166 ± 73 |
|  | p-value (edge *vs*. center) | 0.3902 | 0.2519 | 0.2162 |
| DOPC+Ca | E at edge | 173 ± 126 | 371 ± 313 | 243 ± 246 |
|  | E at center | 348 ± 375 | 917 ± 601 | 492 ± 477 |
|  | p-value (edge *vs*. center) | 0.1929 | 0.0249 | 0.1722 |
| DOPC+PBS *vs*. DOPC+Ca | p-value at edge | 0.4005 | 0.0556 | 0.1355 |
|  | p-value at center | 0.1584 | 0.0046 | 0.0607 |


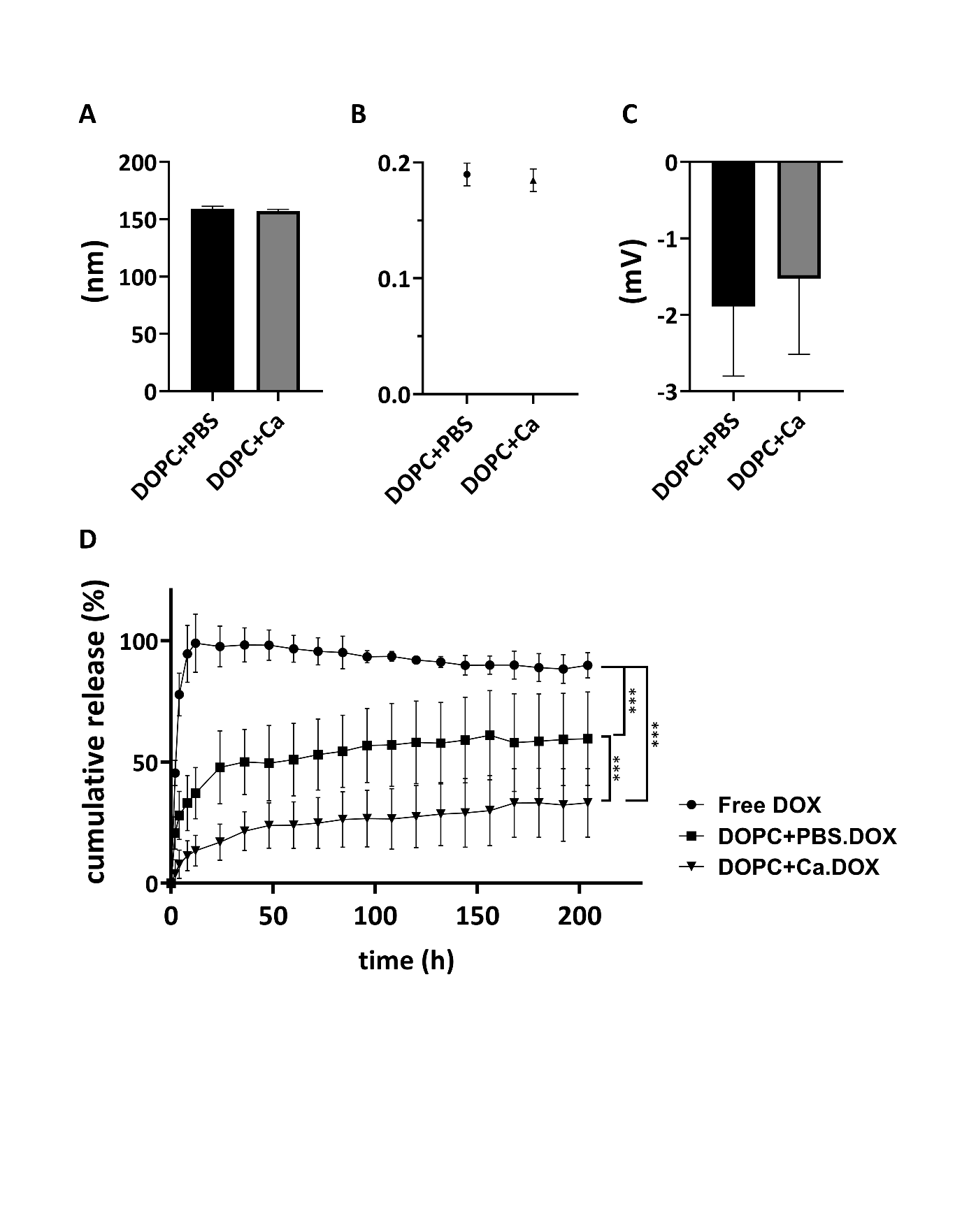


**Figure S1.** Characteristics of lipid nanoparticles (LNPs) determined using dynamic light scattering (DLS; A–C) and drug release kinetics (D). (A) Average diameter, (B) polydispersity index (PDI), and (C) zeta potential of LNPs with different elasticities (*n* = 40). Error bars present SD. (D) Cumulative release (CR) of doxorubicin from each LNP type over the observation period. Results represent mean ± standard error of the mean (SEM) (*n* = 3). Statistical significance was determined using a two tailed Student’s *t*-test; *** p < 0.005


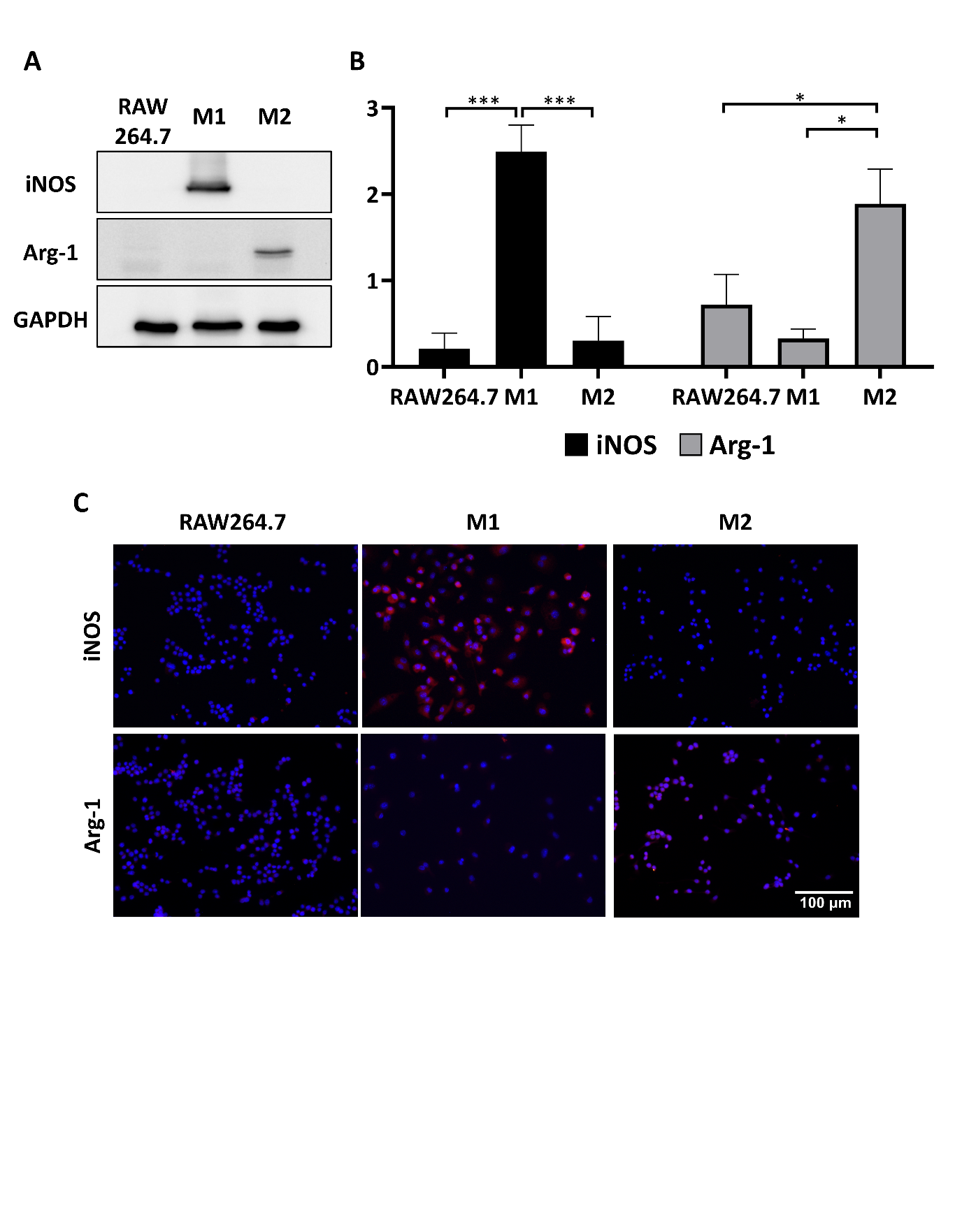


**Figure S2.** Macrophage polarization was confirmed using western blotting (A, B) and fluorescence microscopy (C). (A) Elevated expression of inducible nitric oxide synthase (iNOS) at M1 and arginase 1(Arg-1) at M2 confirmed successful polarization. (B) Relative protein expression of iNOS and Arg-1 calculated from the western blot data (*n* = 3). Statistical significance was determined using a two-tailed Student’s *t*-test. Error bars represent standard error of the mean (SEM); *p < 0.05, ***p < 0.005. (C) Fluorescence images showing enhanced expression of iNOS at M1 and Arg-1 at M2


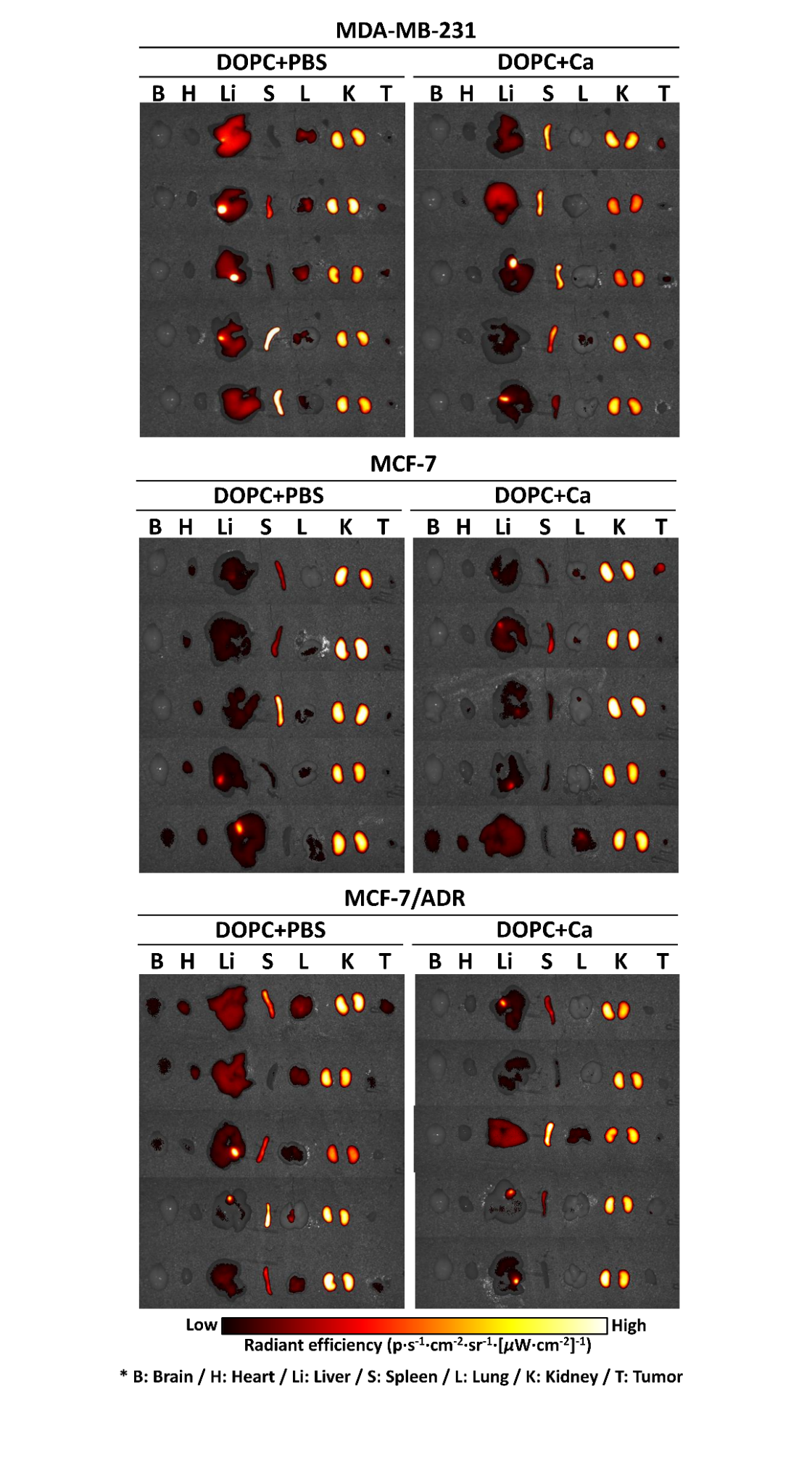


**Figure S3.** The fluorescence images obtained after the completion of the mouse experiments show the fluorescence expression in the tumors, brain, heart, liver, spleen, lungs, and kidneys according to the DOPC+PBS and DOPC+Ca groups in different breast cancer cells. (*n* = 5)


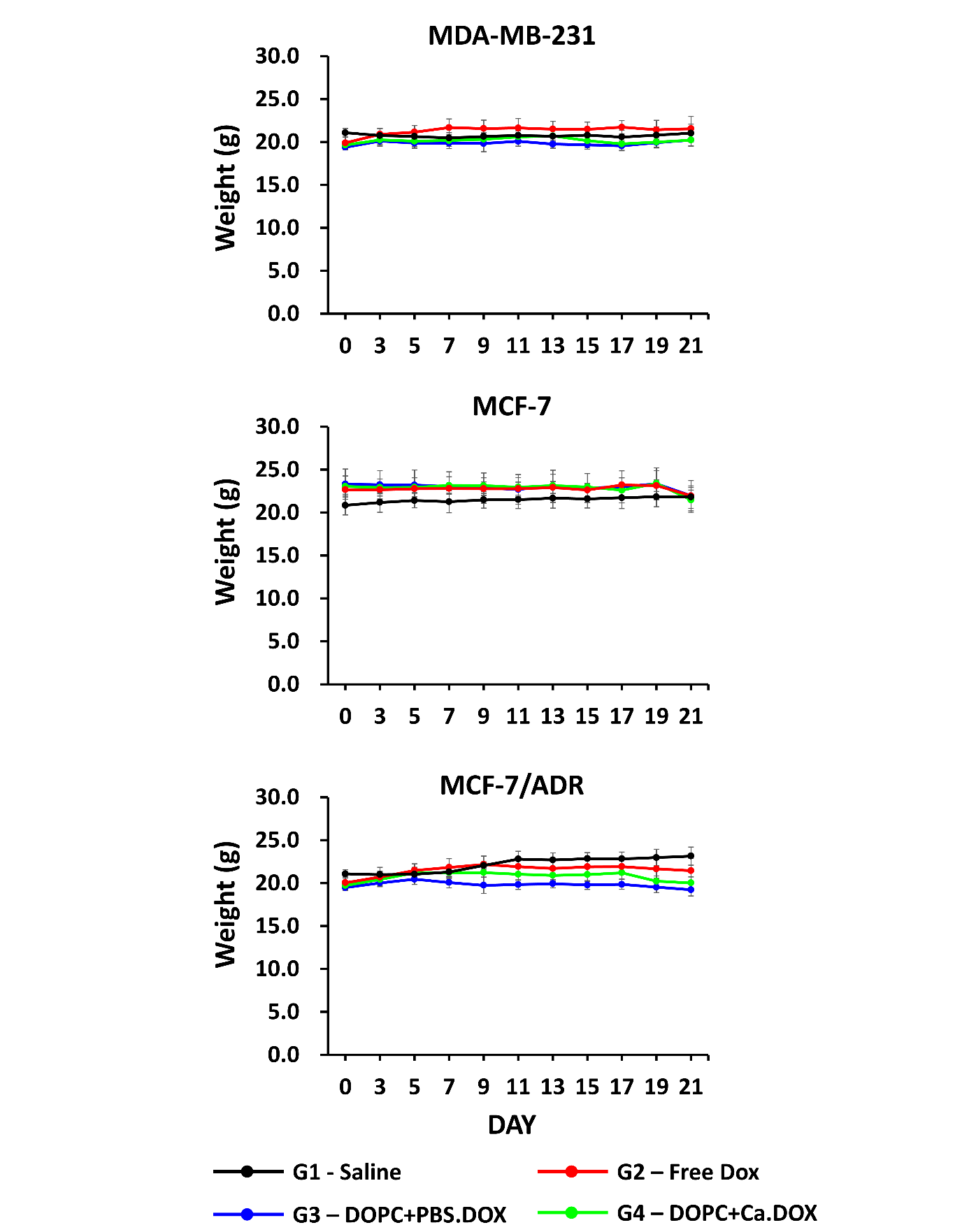


**Figure S4.** Consistent body weights of mice in each group after drug administration. Results represent mean ± standard error of the mean (SEM). (*n* = 5)
